# A Review of Radio Frequency Based Localisation for Aerial and Ground Robots with 5G Future Perspectives

**DOI:** 10.3390/s23010188

**Published:** 2022-12-24

**Authors:** Meisam Kabiri, Claudio Cimarelli, Hriday Bavle, Jose Luis Sanchez-Lopez, Holger Voos

**Affiliations:** 1 Interdisciplinary Center for Security Reliability and Trust (SnT), University of Luxembourg, L-1855 Luxembourg, Luxembourg; 2 Department of Engineering, Faculty of Science, Technology, and Medicine (FSTM), University of Luxembourg, L-1359 Luxembourg, Luxembourg

**Keywords:** robot localisation, UAV localisation, 5G NR, localisation algorithms

## Abstract

Efficient localisation plays a vital role in many modern applications of Unmanned Ground Vehicles (UGV) and Unmanned Aerial Vehicles (UAVs), which contributes to improved control, safety, power economy, etc. The ubiquitous 5G NR (New Radio) cellular network will provide new opportunities to enhance the localisation of UAVs and UGVs. In this paper, we review radio frequency (RF)-based approaches to localisation. We review the RF features that can be utilized for localisation and investigate the current methods suitable for Unmanned Vehicles under two general categories: range-based and fingerprinting. The existing state-of-the-art literature on RF-based localisation for both UAVs and UGVs is examined, and the envisioned 5G NR for localisation enhancement, and the future research direction are explored.

## 1. Introduction

Ground and aerial robots are essential for automating diverse civilian and commercial applications, such as search and rescue operations, connected and autonomous driving, precision agriculture, and road traffic management, among others [1,2]. For example, in the context of new communication technologies, drones may be effectively used as mobile 5G base stations for special events gathering crowds and solving network congestion. Nevertheless, in many of these scenarios, an autonomous robot navigates in a highly dynamic environment and requires precise self-location awareness to operate safely. Thus, localisation can be regarded as the core of autonomous navigation systems. Common localisation systems heavily depend on a Global Navigation Satellite System (GNSS), such as the Global Positioning System (GPS). While it provides sufficient accuracy in most situations, GPS-denied areas might impede or discourage reliance on this technology. Due to the weakness of the GPS signal in indoor environments or forests, cameras, Inertial Measurement Units (IMU) and Light Detection and Ranging (LIDAR) are the principal alternatives to provide valuable information for enabling robot autonomous navigation, which are backed by mature theoretical studies and with many developed solutions. However, each of those sensors presents characteristics that make them unsuitable for specific environments or situations. Vision-based methods provide poor performance in low light, adverse meteorological conditions, or in the presence of visual aliasing. IMU-based localisation suffers from noisy measurements, and motion estimates drift rapidly. LIDAR provides the richest and most precise measurements of the surrounding 3D environment. However, it requires high power consumption when processing large-point cloud data. Moreover, in a wireless sensor network (WSN) with many nodes, GPS is not a reasonable solution regarding cost, power consumption, and form factor requirements of small Internet of Things (IoT) devices [3,4,5,6]. Therefore, multiple and diverse sensors must be integrated and fused to make the system robust.

As a complementary solution, a radio frequency (RF) signal can boost localisation accuracy and robustness. Most RF metrics that have been used extensively include Time of Arrival (TOA), Time Difference of Arrival (TDOA), Angle of Arrival (AOA), and Received Signal Strength (RSS). Different technologies that can provide these measurements include Wi-Fi, Bluetooth, Ultra-Wideband (UWB), Zigbee, Radio Frequency Identification Devices (RFID), Long-Range Radio (LoRA), and cellular networks [7,8]. Cellular networks are beneficial because we can take advantage of the existing infrastructure, which covers many areas. 5G NR has great potential for enhanced localisation, not only providing accurate measurements and tools based on which even centimetre-level accuracy can be expected, but also enabling the implementation of demanding fusion-based algorithms to compensate for the deficit of each sensor measurement through edge computing. The benefits of 5G NR for localisation include (but are not limited to): wide area coverage, high angular and time resolution measurement for localisation, increased probability of Line of Sight (LOS), and a fast data rate. Concentrating on UAVs and UGVs, 5G can revolutionize localisation by providing:

- Edge computing.
- Vehicle to Everything communication (V2X).
- Beamforming.
- Multi-array antenna.

Several existing surveys exist in the area of localisation covering the topic from different points of view, but they often overlap with each other [7,8,9,10,11,12,13,14,15,16,17]. For instance, Dwivedi et al. [15] explore the positioning in 5G networks from the communication point of view. Yang et al. [9] review the positioning based on RF signal for UAVs, with emphasis on the technologies and challenges, and Maghdid et al. [8] focus on the IoT technologies and performance metrics. Chowdhury et al. [10] provide a comprehensive survey for localisation in WSN, which investigates the techniques and algorithms under diverse categories. Kumari et al. [13] narrowed down the review to the localisation in 3D space. Tabassum et al. [12] present a brief overview of the localisation approaches in WSN. Khelifi et al. [11] review the localisation for IoT under various categories of approaches. The reviews and the covered topics are compared in Table 1.

Hence, this survey aims to address the issue that remained untouched by other state-of-the-art reviews. As mentioned, the existing studies focus on specific aspects of localisation, such as technologies, methods, IoT applications, communication perspective, and classification of the approaches. However, this review covers the full extent of RF-based localisation approaches, first by distinguishing the type of information that can be decoded from the received signal in the form of features and then by categorizing the methodologies based on the use, whether range based or fingerprinting (data-driven), of these RF features. Additionally, we address the use of RF for UAVs and UGVs applications and discuss the future potential of 5G. Therefore, the main contributions of this review paper are as follows:

- Investigating the algorithms for RF-based localisation that are highly likely to be used for challenging UGV and UAV applications.
- Reviewing the existing works considered using RF specifically for UAVs and UGVs positioning.
- Discussing the new potential that 5G NR will provide to cope with the current issues in UAVs and UGVs localisation problem.
- Discussing the challenges for ground and aerial robots localisation and its integration with 5G NR.

The survey paper is organized as follows: After introducing the main RF features used for localisation, we briefly introduce localisation techniques and how they can be used. Then we proceed to explore the up-to-date, state-of-the-art algorithms that have been widely used in RF-based localisation. In the next section, we concentrate on the available RF-based localisation applied to UAVs and UGVs. The 5G NR potentials and benefits expected for the aerial and ground vehicles localisation are also explained. We also review the current state-of-the-art localisation based on 5G NR. Finally, the challenges and future direction are be explored.

## 2. RF Features

The received radio frequency signal involves features that encode information about the transmitter’s and receiver’s relative position. Therefore, in this section, we describe the features that are most relevant and widely used for localisation purposes.

### 2.1. Received Signal Strength

Received Signal Strength (RSS) is one of the features most frequently used in localisation due to its low hardware requirements and easy implementation compared to its counterparts. RSS is the received power in the receiver over the bandwidth. The main concept behind using RSS for localisation is that the power attenuation of the signal from transmitter to receiver depends on the distance. Extraction of the exact relation, however, in real environments seems infeasible due to the unknown channel model, and the majority of existing literature relies on using simple models for mapping RSS to distance or range. The most popular model is the log-distance one-slope propagation model:

(1) $$\begin{array}{cc} P_{d}\left[dB\right]= & P_{0}\left[dB\right]-10\alpha log_{10}\left(d/d_{0}\right)+X_{\sigma }\left[dB\right]+b\left[dB\right]\;,\\ d= & ||X-S||\;, \end{array}$$

where:

- $P_{0}$: power at the reference distance $d_{0}$ from the transmitter (usually 1m).
- $P_{d}$: received power at distance *d* from the transmitter.
- $X_{\sigma }$: shadowing effect( mostly considered as Gaussian).
- α: Path loss exponent (PLE), the rate at which power decrease over distance.
- *b*: bias error.

A more complex form of propagation model has been used, such as two-slope, third-order, and higher up to the sixth-order model [18,19]. Lee et al. [20] propose finding the best model for each transmitter–receiver pair using the Genetic algorithm to search between multi-state path loss models with k states. It should be noted the transmission power is assumed to be fixed. When power control is applied at the transmitter, which might be the case in 5G, range inference based on distance is impossible. Transmission power and PLE are usually obtained by conducting a pre-test, collecting data from the environment, and matching the suitable values based on the model. Algorithms that deal with unknown PLE and transmission power thus offer the advantage of removing the need for an intensive pre-test phase.

Another contributing factor for less accurate RSS-based localisation—in addition to channel modelling error—is that RSS is not stable and fixed over time and under changing environments. In [21], three main factors that play an important part in unstable RSS readings are discussed, and the effects of antenna orientation, foreground obstacles, and beacon density are explored.

### 2.2. Time of Arrival

The Time-Of-Arrival (TOA) technique measures the time it takes for an RF signal to travel from transmitter to receiver, multiplying by the signal’s speed in the medium, which is usually the propagation speed of light. Thus, the range can be inferred. As the obstruction does not significantly impact the speed of the wave, TOA delivers higher accuracy compared to RSS provided that tight clock synchronisation among receiver and transmitters is carried out, and there is a Line-of-Sight (LOS) path. The existence of LOS and clock synchronisation, however, are the two challenges in TOA estimation. When no LOS path exists, the multi-path components are received while each travels further than the distance between the receiver and transmitter. Stringent clock synchronisation also calls for complex hardware.

### 2.3. Time Difference of Arrival

Time Difference Of Arrival (TDOA) is another time-based measurement which illustrates the difference between the time signal travelling from two transmitters to the same receiver. In the conventional approach, cross-correlation is used to extract this value, computing the delay that maximizes the cross-correlation function. One advantage of TDOA compared to TOA is that synchronisation is only required among the transmitters. TDOA suffers from the same issues as TOA, i.e., imperfection hardware and LOS blockage.

### 2.4. Angle of Arrival

The Angle-Of-Arrival (AOA) method for localisation has not been incorporated as much as its counterparts. The use of directional antennas, multi-element arrays for MIMO, and mmWave, especially for 5G cellular networks, has attracted more attention. Similar to TOA, AOA also suffers from LOS blockage. Other multi-path components from NLOS can lead to erroneous and misleading information about the direction of arrival. Thus, AOA is usually fused with other data for localisation purposes.

### 2.5. Channel System Information

Compared to the mentioned RF features, Channel System Information (CSI) provide much richer information, such that all the other values can be extracted by analysing it. CSI provides information about the communication channel, e.g., fading, scattering, and power decay, and how the propagating signal is impacted at a specific carrier frequency at each path. The CSI amplitude and phase are dependent on the poses of the transmitter, receiver, and the surrounding environment with objects therein. Compared to RSS, TOA, TDOA, and RSS, which provide one value per measurement, CSI consists of a matrix, with each entry representing the Channel Frequency Response (CFR). Thus, there are much more data to deal with and analyse. Moreover, it is raw information that is not particularly intuitive compared to the other measurement. Hence, machine learning algorithms and fingerprinting are suitable candidates for CSI applications.

## 3. Overview of RF-Based Localisation Techniques

Loosely speaking, localisation based on radio signals can be divided into range-based and fingerprinting methods.

- Range-based techniques: Localisation is achieved by inferring the distance or angle of the target from a node based on the measurements. Time Of Arrival (TOA), Time Difference Of Arrival (TDOA), and Received Signal Strength (RSS) provide ranges, while Angle Of Arrival (AOA) provides bearings measurements. In a sensor framework, two or several of these methods can be combined, which might result in a better outcome. In the next stage, the extracted ranges or bearings are used to estimate the location, taking advantage of various mathematical tools such as Maximum Likelihood (ML), the Least Squares (LS) approach, the Bayesian model, or different types of filters such as the Kalman filter (KF), extended Kalman (EKF), Unscented Kalman filter (UKF), and Particle filter (PF). In the next section, we will explain the methods used for range-based localisation.
- Range-free or Fingerprinting: Instead of calculating the distance or direction, the environmental survey is performed to obtain fingerprints or features recorded on a database, such as the location-RSS pair’s value, and then in online mode, for every new measurement, the localisation is performed by finding the best match in the data set. More generally, this method consists of mapping and matching. Compared to the range-based approach, fingerprinting techniques are more accurate and demanding to implement, requiring a pretest to create an extensive database. In addition, fingerprinting methods differ in generating and updating the data set and the matching process. Nevertheless, fingerprinting is widely used for CSI and RSS-based localisation.

## 4. Range-Based Algorithms

Having extracted ranges or bearing from the RF signal, intuitive mathematical or geometrical approaches are then leveraged to infer the receiver’s position. The most important methods include Multi-Lateration/Triangulation, Min-Max, Multidimensional scaling (MDS), Least Squares (LS), Maximum Likelihood (ML), the Bayesian inference method, and Bayesian Filters. Table 2 provides a comparison of range-based methods.

### 4.1. Multi-Lateration/Triangulation

**Figure 1:**
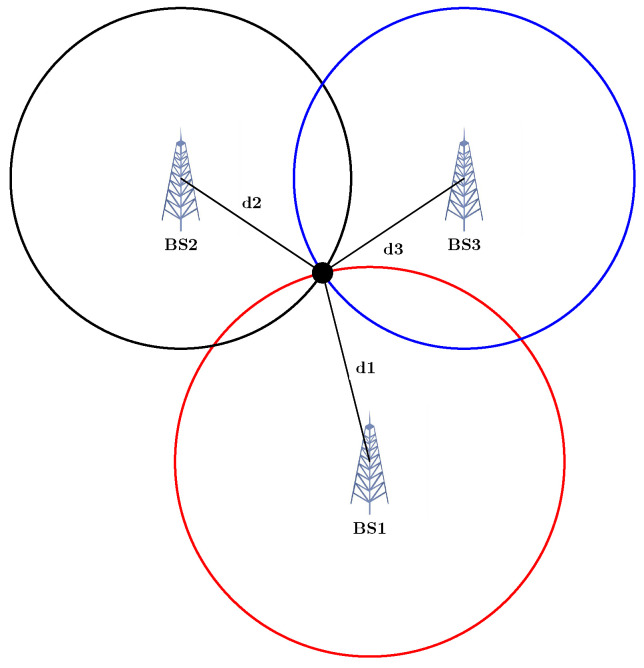
Trilateration: localisation based on the range from 3 anchors (TOA, RSS).

**Figure 2:**
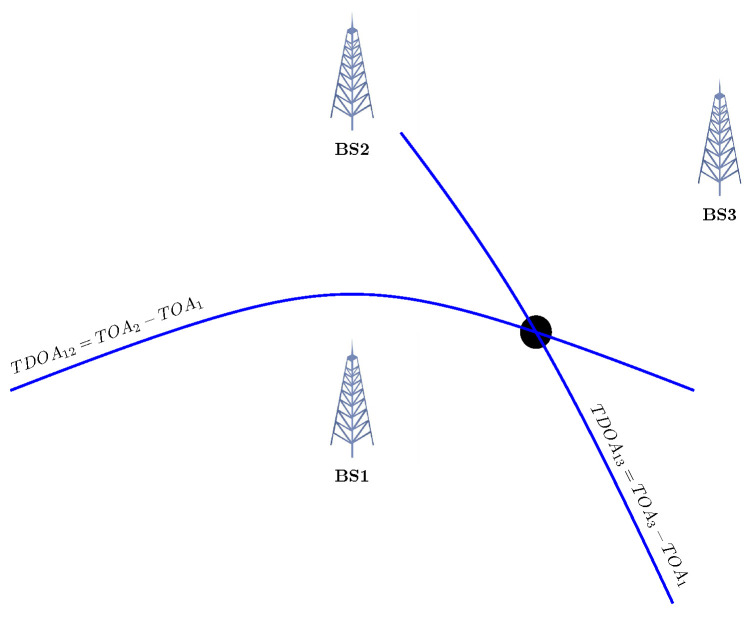
Localisation based on TDOA, the intersection of the hyperbolas.

**Figure 3:**
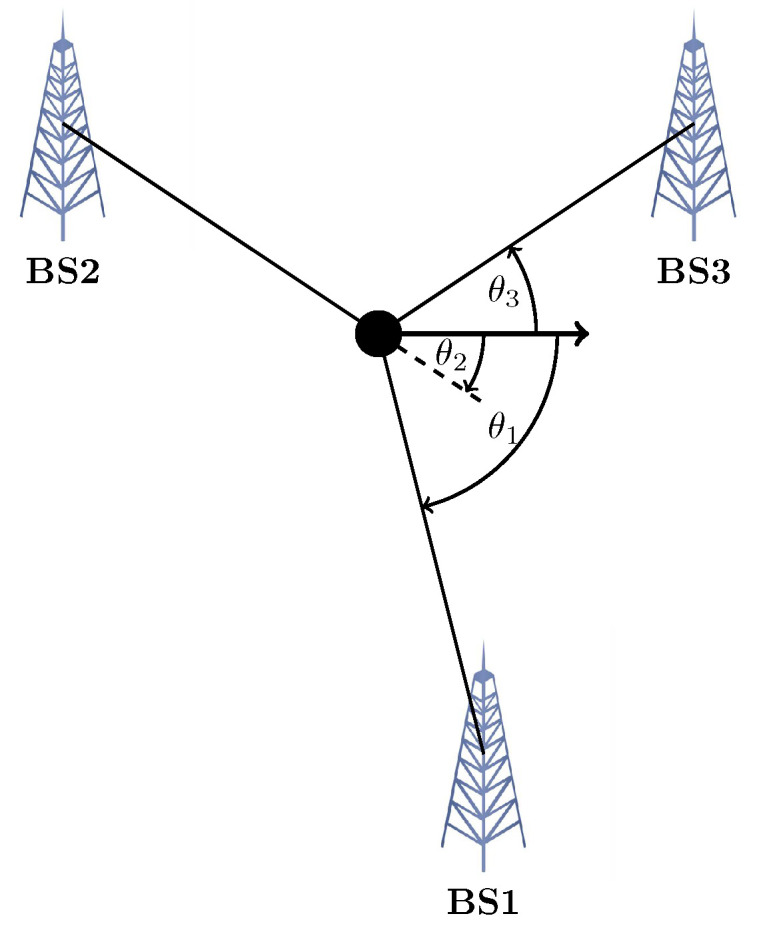
Triangulation: localisation based on AOA from 3 BS.

Based on the ranges, angles, or range differences, a set of equations can be constructed which usually result in an over-determined set of non-linear equations. For N number of BS position at $S_{i}={\left[S_{ix},S_{i_{y}}\right]}^{T}$ and the target at location $X={\left[X_{x}X_{y}\right]}^{T}$, we can write

(2) $$\begin{array}{cc} d_{i} & =||X-S_{i}\left|\right|+\sigma ,\;i=1,2,\ldots ,N\;, \end{array}$$

(3) $$\begin{array}{cc} \delta d_{i} & =||X-S_{i}-S_{1}\left|\right|+\sigma ,\;i=2,...,N\;, \end{array}$$

(4) $$\begin{array}{cc} a_{i} & =\arctan(\frac{X_{y}-S_{iy}}{X_{x}-S_{ix}})+\sigma ,\;1,\ldots ,N\;, \end{array}$$

where σ is the measurement error, while $d_{i}$, $\delta d_{i}$, and $a_{i}$ are the range, range-difference, and angle measurements. Geometrically speaking, in the simplest scenario, having the range from target to three Base stations (BS) in 2D, the intersection of three circles with the centre of the BS and radius of the measured range will be the solution (see Figure 1). In 3D, the intersection of four Spheres is the response. For TDOA, the hyperbolas can be constructed, with the focus being the two BS (see Figure 2). In the AOA framework, the angles measured with the help of the geometric properties of the triangle could determine the target location (see Figure 3). In an ideal case, these methods intersect in one single point. However, this never happens in the real world, which gives rise to several scenarios [22].

The set of equations can then be solved either in approximated closed-form or iteratively. Closed-form formulation leads to an easy, low-complexity solution. For example, in [23], a closed-form algebraic solution for target localisation for both trilateration (three reference points) and multi-lateration (more than three reference points) is solved. The uncertainty of each information piece is considered by adding a variance matrix to the equations. However, it assumes the known covariance matrix error, which is not realistic. In an extended version of this method [24], after applying the standard multi-lateration procedure, if the solution lies within the reference node positions, it is considered the final solution. Otherwise, the algorithm keeps searching for the solution inside the zones that are determined within the reference nodes based on the strength of the RSS level (the zone closer to the base station from which the strongest RSS level is read has the highest probability of containing the solution). In the search process for a solution inside a zone, virtual positions are defined inside zones, and then the final target location is selected based on the distance between the first estimation and the virtual positions.

### 4.2. Min–Max

**Figure 4:**
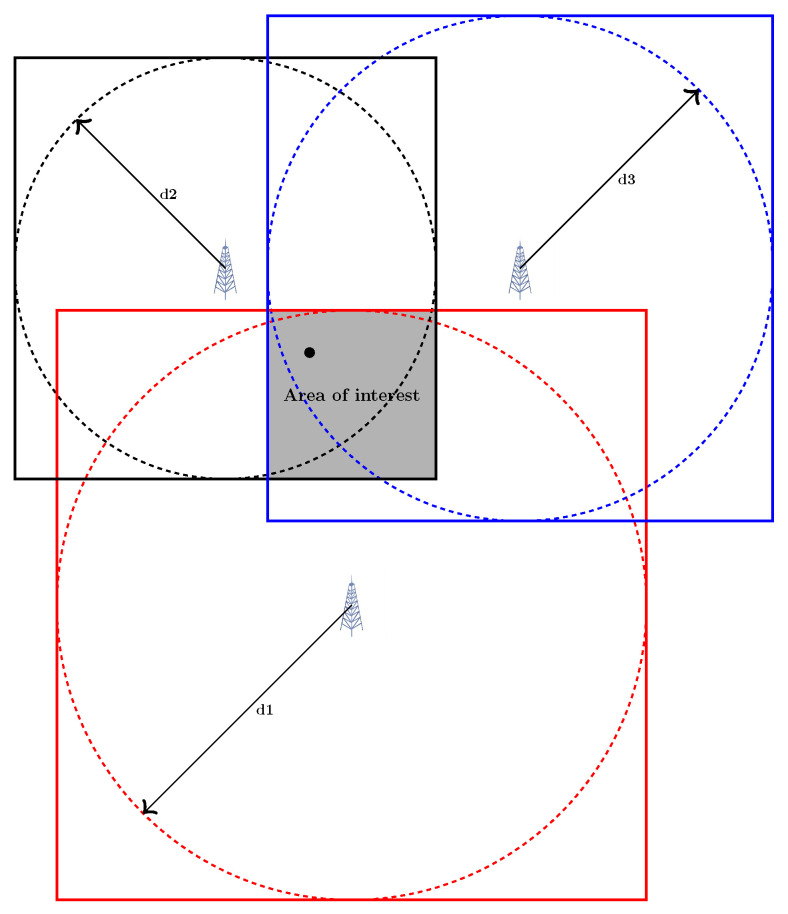
Min–Max algorithm.

Min-Max is a simple, intuitive, and geometrical-based technique when an easy implementation is desired [25,26,27]. According to ranges, squares are formed that circumscribe the circles around each BS with radius $d_{i}$ as the distance between the BS and target. Then, the vertices of a rectangle known as the area of interest are found, as shown in Figure 4. In the simplest version, the centroid of this rectangle is selected as the estimated position. Among Min–Max variants, Extended Min–Max [28] uses the weighted centroid instead of the geometric centroid. Furthermore, Yang et al. [26] introduce a new strategy with a partition area.

### 4.3. Multidimensional Scaling (MDS)

Multidimensional scaling (MDS) is a computationally efficient approach, especially for a cooperative framework consisting of a group of nodes for localisation for which, due to high dimensional search space, finding the optimised value is demanding. MDS is a visualisation technique by which the pairwise range could be mapped to the lower dimensional Cartesian space that can be graphically displayed. The MDS method offers an analytical closed-form solution which makes it advantageous in terms of computational burden, efficiency, and ease of implementation [29,30].

### 4.4. Least Squares (LS)

Least squares (LS) is a well-known method used to estimate an unknown. It can be formulated by finding a solution that minimises the square of the error, such that:

(5) $$\begin{array}{cc} \; & \underset{X}{\arg\;\min}\sum_{i=1}^{N}{\left(RSS_{i}-\left(P_{0}^{i}-10\alpha \log_{10}(||X-S_{i}\left||\right)\right)\right)}^{2}\;, \end{array}$$

(6) $$\begin{array}{cc} \; & \underset{X}{\arg\;\min}\sum_{i=1}^{N}{\left(TOA_{i}c-||X-S_{i}\left|\right|\right)}^{2}\;, \end{array}$$

(7) $$\begin{array}{cc} \; & \underset{X}{\arg\;\min}\sum_{i=2}^{N}{\left(TDOA_{i}c-||X-S_{i}\left|\right|+||X-S_{1}\left|\right|\right)}^{2}\;, \end{array}$$

(8) $$\begin{array}{cc} \; & \underset{X}{\arg\;\min}\sum_{i=1}^{N}{\left(AOA_{i}-\arctan\left(\frac{X_{y}-S_{yi}}{X_{x}-S_{xi}}\right)\right)}^{2}, \end{array}$$

where *c* is the speed of the light, $RSS_{i}$, $TOA_{i}$, $TDOA_{i}$, and $AOA_{i}$ are the measurements with respect to the ith BS. For the cooperative case where there are *M* targets to be localised, it can be written as:

(9) $$\begin{array}{cc} \; & \underset{X}{\arg\;\min}\sum_{j=1}^{N}\sum_{i=1}^{M}{\left(RSS_{ij}-\left(P_{0i}-10\alpha \log_{10}\left(|\right|X_{j}-S_{i}\left||\right)\right)\right)}^{2}, \end{array}$$

(10) $$\begin{array}{cc} \; & \underset{X}{\arg\;\min}\sum_{j=1}^{N}\sum_{i=1}^{M}{\left(TOA_{ij}c-\left|\right|X_{j}-S_{i}\left|\right|\right)}^{2}, \end{array}$$

(11) $$\begin{array}{cc} \; & \underset{X}{\arg\;\min}\sum_{j=1}^{N}\sum_{i=1}^{M}{\left(TDOA_{ij}c-\left|\right|X_{j}-S_{i}\left|\right|+\left|\right|X_{j}-S_{1}\left|\right|\right)}^{2}\;, \end{array}$$

(12) $$\begin{array}{cc} \; & \underset{X}{\arg\;\min}\sum_{j=1}^{N}\sum_{i=1}^{M}{\left(AOA_{ij}-\arctan\left(\frac{X_{yi}-S_{yi}}{X_{xi}-S_{xi}}\right)\right)}^{2}\;. \end{array}$$

A straightforward solution to cope with the highly non-linearity is linearisation, such as Taylor expansion [31]. However, the recursive LS method offers more fidelity and accuracy in the cost of complexity and computation burden [31]. The Weighted Least Squares (WLS) method is more efficient, which puts different weights on the measurement. In one intuitive method, weights are selected based on the distance. The optimal method is to exploit the covariance of the measurement noise for WLS. When such information is not at hand for noise, there are some efforts to estimate it [32].

In addition to position, other unknown parameters can also be jointly estimated, such as unknown PLE [33] and transmission power (for RSS-based localisation) [34]. Unfortunately, [34] also considers the unknown weight matrix and Non-line-of-sight (NLOS) impact by adding a random variable to the propagation model, which adds to the complexity of the problem. To deal with this issue, the semi-definite relaxation (SDR) technique is used as an approximation, transforming the problem into convex semi-definite programming.

In a more efficient framework, the relative error is employed instead of the estimation error, which is called Least Squares Relative Error (LSRE) [35,36]. The relative error is the ratio of the absolute error to the measured value. In LS, all observations are treated equally, implying the same precision for all data, which is critical in reality.

### 4.5. Maximum Likelihood (ML)

Maximum Likelihood is one of the most used approaches to localisation, resulting in a non-convex, non-linear optimisation problem. The object of ML is to maximise the likelihood function:

(13) $$\begin{array}{c} \underset{X}{\arg\;\max}\;p\left(X|z\right). \end{array}$$

The LS minimisation of the observation errors normalised by measurement variances gives the ML solution.

(14) $$\begin{array}{cc} \; & \underset{X}{\arg\;\min}\sum_{j=1}^{N}\sum_{i=1}^{M}{\left(\frac{RSS_{ij}-\left(P_{0i}-10\alpha \log_{10}\left(|\right|X_{j}-S_{i}\left||\right)\right)}{\sigma_{ij}}\right)}^{2}\;, \end{array}$$

(15) $$\begin{array}{cc} \; & \underset{X}{\arg\;\min}\sum_{j=1}^{N}\sum_{i=1}^{M}{\left(\frac{TOA_{ij}c-\left|\right|X_{j}-S_{i}\left|\right|}{\sigma_{ij}}\right)}^{2}\;, \end{array}$$

(16) $$\begin{array}{cc} \; & \underset{X}{\arg\;\min}\sum_{j=1}^{N}\sum_{i=1}^{M}{\left(\frac{TDOA_{ij}c-\left|\right|X_{j}-S_{i}\left|\right|+\left|\right|X_{j}-S_{1}\left|\right|}{\sigma_{ij}}\right)}^{2}\;, \end{array}$$

(17) $$\begin{array}{cc} \; & \underset{X}{\arg\;\min}\sum_{j=1}^{N}\sum_{i=1}^{M}{\left(\frac{AOA_{ij}-\arctan\left(\frac{X_{yi}-S_{yi}}{X_{xi}-S_{xi}}\right)}{\sigma_{ij}}\right)}^{2}\;. \end{array}$$

Solving it is not trivial and imposes a high computational cost. Subsequently, variant relaxations criteria are used to deal with the complexity, such as semi-definite programming (SDP) relaxation and second-order cone programming (SOCP) relaxation to approximate the complex problem, or numerical approaches such as Newton–Raphson are employed. In a joint-ML scheme, location along with channel parameters are estimated [37,38]. Ref. [38] also explores the combination of multi-lateration with ML to compromise between performance and complexity where multi-lateration is exploited for location estimation, and ML is utilised for channel parameters estimation. The paper then compares it with the joint ML method. The effect of the fault and various noises are taken into account in [39,40]. Ref. [39] addresses byzantine fault and NLOS effect. Byzantine fault is considered by including non-Gaussian interference noise corrupting the transmission data, and the NLOS effect is modelled by adding a bias term to the propagation model. First-order Taylor series expansion is utilised to simplify the model so that some transformation turns it into a generalised trust-region sub-problem (GTRS). Additive and multiplicative noises are handled in [40] using ML, taking advantage of iterative expectation-maximisation.

### 4.6. Bayesian Inference Method

The Bayesian approach gives a distribution, instead of an estimated value, as an outcome which is more informative than LS. Compared to ML, where parameters are considered fixed, in the Bayesian method, they are treated as a random variable with known prior distribution [41]. Based on Bayes’s theorem, the position and the parameters act as random variables where the prior information and observations are leveraged to infer and update the posterior distribution of unknown random variables. For example, in [42], PLE and position are treated as mutually independent random variables from which the posterior distributions are derived. To do so, a message passing algorithm, called belief propagation [43], is used on the factor graph, which allows for efficient computation of marginal distributions and dealing with the problem’s intractability. The cooperative localisation scenario is also dealt with using the Bayesian method in [42]. In this paper, in addition to PLE, transmission power is estimated.

### 4.7. Bayesian Filters

When estimating the dynamic states, which is the case in robotic applications, filters can work effectively. They work by alternating between two steps, i.e., prediction and update. For example, the classic Kalman filter is the optimal estimator for a linear system in the presence of Gaussian noise. They can also be employed readily to fuse the observations of various sensors. In robotics, the movement dynamic is considered as constant velocity or constant acceleration, which usually performs acceptably in practice.

While the process model is linear, the observation model becomes non-linear. In this case, sub-optimal non-linear filters are exploited. These include the Extended Kalman filter [44], Particle Filter [45], and Unscented Kalman Filter [46]. The Extended Kalman filter is the first-order Taylor series expansion of the non-linearity widely used in diverse applications with an acceptable outcome. However, such a first-order approximation might introduce errors in the posterior distribution estimation [47]. The unscented Kalman filter (UKF) and particle filter (PF) can outperform EKF by removing the linearisation step and carrying out deterministic sampling. Based on unscented transform (UT), UKF transforms states to weighted sigma points based on which prediction and update are executed. PF performs sequential importance sampling, drawing the particles and their corresponding weights from the probability density. We will review works based on Bayesian filters in Section 7.

## 5. Fingerprinting

As an alternative to the range-based approach, fingerprint methods are based on the collected data to infer the position instead of relying on the model. This method can better handle the error caused by the modelling errors and noises. Fingerprinting is mostly used for RSS and CSI-based localisation, but it has also been exploited for other RF-based measurement [48,49,50,51,52] and for a hybrid scheme [53,54,55].

**Figure 5:**
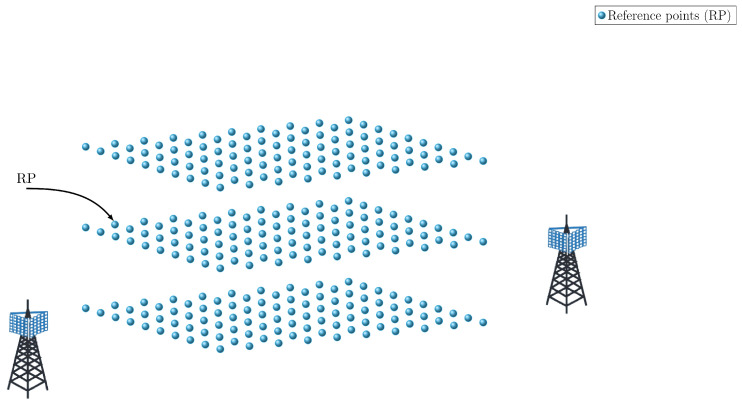
Reference points in 3D.

**Figure 6:**
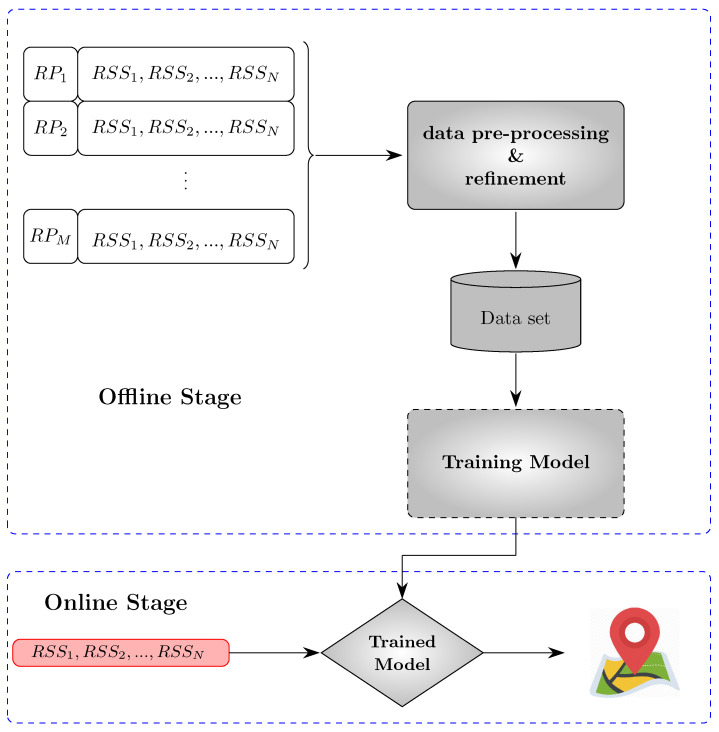
Fingerprinting block diagram.

This method usually results in more accurate estimation and comes at the cost of the laborious step of data collection. Generally speaking, fingerprinting consists of an offline mode and an online mode. In the offline step, a dataset consists of recorded measurements or RF features, and the ground truth position at references points (RP) (see Figure 5), which are called fingerprints, is generated. Based on this, in the online phase, estimation is made through matching (see Figure 6).

### 5.1. Offline Step

One of the main challenges in the fingerprinting approach is collecting and maintaining a proper fingerprint database. The most straightforward approach to generating the radio map or dataset is to measure and record the fingerprints over all areas. However, due to the ample space, it is not practical to obtain detailed measurements and conduct costly site surveys. Moreover, the fingerprint map becomes outdated due to the radio signal features and dynamic environment. This problem is discussed in [56], where the effect of an incomplete dataset is explored by applying several interpolations and extrapolations to recover the missing data. The work of [57] explores deterministic adaptive path loss model interpolation for radio map generation, focusing on improving the speed of the process and decreasing the time of radio map construction. Each AP’s RSS path loss model is extracted using available reference points, LS, and interpolation. Gaussian regression is another probabilistic method used for this purpose. The authors of [58] exploit Gaussian Process Regression (GPR) to predict the RSS spatial distribution based on the available dataset. To achieve this, instead of using a basic zero mean function and a single squared exponential as kernel function for GPR, compound GPR kernel functions are used. In [59], more information regarding radio map generation techniques can be found. Experiments in the real world also make this comparison.

It is worth mentioning that most fingerprinting localisation exploits the collected raw data or their scaled value. However, this is not efficient in the presence of heterogeneity. The heterogeneity of device pairs, i.e., transmitter and receiver, can affect the RF-based localisation, as each device uses different hardware parameters, such as antenna gain for transmitters. The different signatures might be recorded in the same situation for each transmitter-receiver combination. To remedy this issue, other variants of perceived measurements are used. For example, differences in signal strengths are used in [60] to address both device heterogeneity and temporal variation of RSS. Another differential RSS-based localisation is handled in [61]. Finally, the authors of [62] present hyperbolic location fingerprinting in which fingerprints are recorded as RSS log ratios between pairs of base stations instead of absolute RSS.

### 5.2. Online Phase

Machine-learning algorithms are mainly used for matching and estimation, including K Nearest Neighbor (KNN), Weighted K Nearest Neighbor (WKNN), Artificial Neural Network (ANN), Convolutional Neural Network (CNN), Support Vector Machine (SVM), and Random Forest.

#### 5.2.1. Classical Machine Learning

K-Nearest Neighbor (KNN) is the first simple candidate to select K points in the dataset based on similarity. Considering the computation complexity, the achieved performance for this algorithm is acceptable. In a WKNN, different weights are assigned to measurements based on criteria such as the distance to the AP. For example, in [63], WKNN is employed where, based on the inverse of RSS distance, weights are assigned to the APs. This criterion, however, is not consistent with position distance due to the nature of path loss, which decays logarithmically based on distance (the reader is referred to [64] for more details). To address this issue, [64] introduces a new weighting process and a new distance measure based on the RSS similarity and spatial position.

One disadvantage of traditional KNN is that it is implemented with a fixed number of K. A Weighted Adaptive KNN Algorithm is adopted in [65] to settle this problem. It can choose a variable number of RPs according to both the improved RSS similarity and position proximity. Density-based spatial clustering of applications with noise (DBSCAN) method and Affinity Propagation Clustering (APC) does not require the number of clusters to be pre-determined and is suitable for handling large databases. The authors in [66] show that clustering could reduce noise’s impact in large datasets. Hence, the DBSCAN method is proposed for localisation, where the fingerprints are divided around a centre point based on the density. Li et al. [67] apply APC offline until the algorithm converges to a final clustering after several iterations and message passing among the points (attraction and attribution messages).

Random Forest is a classifier and regression method based on multiple decision trees that enjoy fast training and prediction, which works well with high dimensional data. Thus, it is a suitable candidate for handling large data-set [52,68]. SVM is another classifier engine that can resolve the regression problem. For localisation, examples of multi-class SVM can be seen in [69,70]. To make the fingerprints more separable, [69] exploits a fuzzy kernel that maps the data to the higher dimension space.

#### 5.2.2. Deep Learning

The capability of Neural Networks (NN) to extract the complex input–output relationship is advantageous for fingerprinting. More notably, NN can be used to directly map the fingerprints to the Cartesian coordinates like [71], which incorporates ANN, taking RSS as input and outputting the estimated position in 2D. Deep learning techniques are more powerful than traditional ML techniques. The main advantage of deep learning is that it is capable of handling a great deal of complex multi-dimensional data that are corrupted by noise [72]. Deep learning can also make better use of the parallelism of GPU architecture, which is equivalent to a lower run-time. There are, however, some challenges and disadvantages of deep learning models. First, they require the availability of rich, varied data representative of the problem to learn meaningful relationships between the input and output. Nevertheless, we lack tools to interpret models and understand for which part of the input space this relationship has been correctly learned and where there is a need for more data. This issue is also related to over-fitting, which appears when the network cannot generalize to unobserved data and makes improper predictions based on the training data. Lastly, finding suitable training hyper-parameters is a time-consuming practice.

One of the promising deep learning-based methods is CNN, which has been widely deployed and successfully used for image classification. CNNs in online mode can perform very fast, though they require a meticulous and time-consuming training phase, which still requires further investigation for fingerprinting localisation. In [73], a six-layer CNN classifier is used to learn and predict under the 74 classes. In [74], Stacked Auto-encoder (SAE) is incorporated to reduce the data dimension before feeding them into the CNN classifier for multi-floor multi-building localisation. In a 5G Internet of Things (IoT) test-bed, [75] combine two CNN for localisation. The first one acts as a regressor that estimates the position in 2D, whose output will provide the input for the second neural network performing as a classifier to estimate the 3D location. A Siamese network consisting of two sub-network (convolutional neural network with shared weights) with offline fine-tuning is proposed in [68] to counter temporal changes of RSS, device heterogeneity, and low RSS samples in a lightweight algorithm. In the training phase, an embedding function is learned that uniquely translates the distance and RSS pairs to an embedding. The training is executed so that each sub-network can take an RSS vector as input and estimate the relative distance of the locations corresponding to the RSS pairs. In the online step, the location is calculated as the weighting average of the RPs, based on the probabilities assigned for the embeddings of online RSS using Random Forest.

It is to be noted that these methods can also be combined in a multi-stage process for localisation. For example, practically, the first simple rough estimation is performed to find the sub-area candidate for the target position. Then, a more accurate estimate is delivered by searching only through the selected sub-area. The work of [76] clusters all data on the database based on signal levels involving RSS and directional antenna gain into the database. First, finding the initial rough estimation, the solution is refined with up-sampling until it meets the desired accuracy. The same two-step procedure is performed in [49] for UAV positioning in 3D based on TOA. After coarse localisation, neural network fitting is executed to refine the estimation. Finally, in [54], rough localisation is performed based on TDOA, and the subarea is searched using a deep neural network (DNN) based on RSS.

## 6. Other Taxonomies

Aside from the techniques based on which the works fall under two broad main categories, there are other points of view that RF-based localisation can take into account.

### 6.1. Distributed vs. Centralised

Two approaches can be distinguished based on how the calculation and process are performed. In a centralised fashion, all data are sent to a central node and station where the computation is carried out. While this requires a less complex algorithm than the distributed approach, it requires a powerful processor. In a distributed way, the process and calculations are distributed among all nodes and subsystems, with each node contributing to the final result. This method demands that more adept and complex algorithms be developed. As the number of nodes in WSN grows, distributed processes become more fascinating.

### 6.2. Cooperative vs. Non-Cooperative

Cooperatively, nodes communicate, share, and use the information to/from their neighbouring node. The advantage of the cooperation between nodes has been discussed in terms of Cramer-Rao Lower Bound (CRLB) [77]. On the other hand, non-cooperative methods are more energy efficient and less complex.

### 6.3. Anchor-Based vs. Anchor-Free

In an anchor-based scheme, we have information about the location of some nodes or BS called anchors obtained either via GPS or by manually deploying them. This information and measurement are used as input for the localisation algorithm. In the anchor-free method, however, there is no information about the positions of the nodes. While anchor-based are more accurate, anchor-free approaches are advantageous because they are more scalable and can remove the process of anchor deployment [78,79].

### 6.4. Static vs. Mobile

The majority of localisation algorithms based on RF focused on the static target. The extension of these results to mobile targets, which is mostly the case in robotic applications, is non-trivial. One critical issue in mobile target localisation is real-time feasibility. In the next section, we will review the localisation of mobile robots.

### 6.5. Technologies

Depending on the available infrastructure, desired accuracy, cost, and the environment, different technologies, such as Wi-Fi, Bluetooth, Ultra-Wideband (UWB), Zigbee, Radio Frequency Identification Device (RFID), cellular network, and Long-Range (LoRA) radio, can be exploited. For example, UWB transmits signals across wide bandwidth in a short range, delivering centimetre accuracy, and is suitable for indoor localisation. On the other hand, Wi-Fi benefits from exploiting existing Wi-Fi infrastructure for localisation. However, lower accuracy will be expected. More details can be found in [7].

### 6.6. 2D vs. 3D

A large part of the studies of localisation so far have been addressed in 2D space. Theoretically, it is claimed that most of them can be extended to the 3D case, but in practice, localisation in the vertical axis comes with a much higher error than the x–y axis. Therefore, 3D localisation is worth further research, which will be highly important for drone applications. This issue is discussed more deeply in [13,38].

### 6.7. Performance Parameters

Evaluating the performance of the localisation algorithm is not straightforward. Many criteria need to be considered for comparison: accuracy, precision, complexity, scalability, security, reliability, cost, and stability [8].

## 7. RF-Based Localisation for Aerial and Ground Robots

Most of the RF-based localisation is applied to sensor network systems. What makes these attempts non-applicable to mobile vehicles in a straightforward way are the new challenges that the localisation of UAVs and UGVs will provide.

- UGVs, and especially UAVs, are highly manoeuvrable, with high speed. The existing state of the art for WSN localisation focuses on fixed targets and cannot address the rapid changes in the target location and the real-time implementation.
- The mobility of vehicles calls for a combination of other sensors, such as IMU and Images. The combination of the sensor data, especially images and RF, has not been studied in localisation.
- The majority of current works in WSN often consider just 2D cases, while vertical estimation is of great importance in UAV localisation.
- The accuracy and robustness in demand in UAVs and UGVs localisation applications are more critical. Usually, very accurate estimation is necessary, while in WSN, rather rough estimation suffices. This, for instance, rules out relying merely on RSS, which is the case for most of the existing state-of-the-art RF localisation.
- Use of limited technologies is the other drawback. For robot applications, UWB is used most. It is limited to indoors and is suitable for short range. New Technologies, especially 5G NR, have rarely been considered so far. In 5G, RSS would not be the most relevant feature, so there would be a shift to the use of this technology’s new potentials and capabilities.

Bayesian filters are among the most used methods for mobile robots localisation, Unscented Kalman Filter [80,81], Particle Filter [45,82,83], Extended Kalman Filter [84,85,86,87] and different variants of it, such as Square Root Cubature Kalman Filter (SRCKF) [88] and Diffusion Extended Kalman Filter (DEKF) [89].

Localisation of a UGV moving with constant speed is developed in [85] using TDOA, incorporating an EKF with an adaptive fading factor to update the prediction covariance to account for the divergence issue of EKF. In a specific scenario [84], localisation is performed for a group of five UAVs, one inside the room and the other four outdoors, based on the information gathered from GNSS, IMU, camera, UWB, and Wi-Fi measurement by all the drones. UWB provides range measurements, and WiFi feeds RSS for indoor UAVs. EKF is used to fuse the measurements and jointly update and estimate all UAVs states and covariance considering the six-DOF (degree-of-freedom) model. An event-driven sampling and transmission mechanism used to counteract the effect of RSS volatility is proposed in [88], where the anchors respond to the mobile robot if some conditions on the received signal are met. The work of [86] combines two EKFs to work simultaneously for location estimation based on TDOA. First, the model is augmented by a weighting filter used in one EKF framework to estimate the state. Then, this estimation is used by the other EKF to update the weights. In a decentralised strategy, [89], diffusion Kalman filter and EKF (DEKF) are integrated to estimate a target based on AOA where a group of UAVs based on AOA share their state estimation, as well as Jacobian information.

In [45], PF is incorporated to estimate the position of a robot in 2D space based on RSS. Generating the radio map by collecting training fingerprints, Kernel Density Estimation is included to build the probabilistic observation likelihood for sample importance weights selection. The average of RSS at each point and the variance are recorded in the database. To enhance the accuracy, an adaptive local search is employed to detect and remove unreasonable estimates by limiting the search area. Furthermore, a mechanism is applied to select just the subset of access points with lower variances to reduce the computational burden. The algorithm is tested for four cases with around 1 meter mean accuracy. The author in [90] fuses odometer data and Wi-Fi RSS to track mobile robots using PF. Executing experiment in 2D, decimeter accuracy is delivered. A new particle-filter-based algorithm is introduced in [83] to improve the estimation with fewer particles. To this goal, the traditional sequential prediction and update steps are carried out in one stage through the maximum likelihood estimator. Experimental results with WiFi RSS indoors show improvement, reaching about 0.5 m^2^ means squared error.

For target localisation, a fingerprinting method is explored in [82]. The radio map is continuously updated while collecting RSS and performing the target localisation simultaneously. The database, the anchor positions, and propagation model parameters are stored and updated. Instead of using the log-distance model, a new model based on collected data is fitted. The model consists of three terms: path loss, measurement noise, and multi-path effect. Four simple functions are proposed and tested for the path loss model to select the best one: linear, nonlinear, and two log-distance models with different parameters. The result indicates that the log-distance model is not as accurate as the first two. The best model is of the form $c_{1}d^{\lambda_{1}d}+c_{2}e^{\lambda_{2}d}$. Based on the model extracted, the fingerprints are modelled as the probability density function. In the sequel, a particle filter is employed for target tracking in online mode.

Two complex ML algorithms are reported in [91,92], where simulations are performed for evaluation. The work [91] uses RSS from multiple BS along with trajectory information (velocity) of the drone to localize an UAV with unknown transmission power. A Joint ML is formulated and solved based on the trajectory information and multiple BS. Fixing the positions in the ML function, first, transmission powers are estimated, which are assumed to be constant. The result is then used to optimise the ML functions based on positions, employing an exhaustive grid search. Two low-complexity alternative algorithms are also suggested, where the former ML problem is broken into several parts, which are solved separately. Then, the final result is calculated as the weighted combination of the separate estimations. Notably, constant velocity is assumed at each time step. A different scenario is considered in [92], where multi-UAVs are supposed to localize a fixed passive RF emitter based on the RSS. A group of UAVs is flying and tracking a predefined trajectory, with one at the centre. To optimise the aggregate likelihood function in a distributed manner, second-order Taylor expansion is exploited to introduce a surrogate function, based on which the Min–Max algorithm is applied. In a two-step procedure that is iterated over, a local update is performed on each UAV based on the centre UAV estimation. In the fusion step executed on the centre UAV, all UAVs share their local estimation with the centre UAV for fusion to generate a new estimation and distribute it among all other UAVs. To reduce the communication overload, an alternative approach is proposed requiring one round of communication, i.e., edge UAVs transmit their information to the centre UAV. In the centre UAV, all estimations are linearly fused with weights approximated based on the Fisher information matrix. On simulations, the authors show that this algorithm improves the result in terms of the root of mean square error (RMSE) in cost of complexity. Another MLE-based method is suggested in [93] based on UWB time-of-flight, in which non-convexity is handled by linearisation. Moreover, the geometry configuration of the anchors and how it impacts the localisation is investigated in terms of CRLB, while both simulations and experiments are conducted.

Simultaneous Localisation and Mapping (SLAM) using multiple robots are explored in [94] leveraging Wi-Fi fingerprinting and odometry in 2D. A graph is constructed with nodes as robots pose and constraints including odometry, individual RSS fingerprints, and similarity of RSS fingerprints between robots. For the trajectory optimisation using graph-based SLAM, the distance of fingerprints and the variance is derived from the database using a simple model.

Instead of a conventional deterministic propagation model, [95] uses a probabilistic likelihood function with the aid of symmetric trapezoidal distribution over discrete localisation grids. Tethering (in which a mobile node localizes, tracks, and follows another mobile node) is performed by integrating RSS and odometry information. The 2D area is divided into grids, with discrete probability. Having received a new RSS value, a Bayesian update is used to update the probability of the grids and achieve full posterior probability distribution over all grids, based on which the current location is extracted. In a very recent paper, [96], the performance of AOA for UAV localisation in a cellular network is investigated, and accuracy of fewer than 45 meters is reported.

Stojkoska et al. [97] incorporate Multi-dimensional Scaling (MDS) and Weighted Centroid Localisation (WCL). Al-Jazzar and Jaradat [98] introduce a geometrical approach. Based on six AOA of sensor doublets, the UAV position is found through mathematical techniques as the intersection of six. The LS method is employed in [99] for UAV localisation in 3D fusion of AOA and TDOA. Nguyen et al. [100] explore relative localisation, estimating the location of the UAV with respect to a target. Relative localisation is useful for formation control [101] and autonomous docking. This paper fuses UWB range measurements with vision-based data. Employing recursive LS, decimeter accuracy is acquired in a 2D experimental setup. The mentioned methods are compared in Table 3.

The existing state-of-the-art literature on robot localisation is limited to very specific scenarios, technology (UWB), RF features, and sensor data. In more detail, two main limitations have to be addressed:

- Limited to specific technologies and sensor data: Most papers use RSS due to its easy-to-use hardware. In that case, acceptable accuracy is achieved by using UWB, which is limited for indoor use with short range. TOA-based localisation is also achieved mostly by taking advantage of UWB. Moreover, many possibilities are missing in the literature, such as the integration of images, and LIDAR with RFs.
- Limited Accuracy: accuracy is one of the main concerns in UAVs and UGVs localisation. Only relying on simple algorithms and sensor data, like RSS, might not be an appropriate solution, especially with the upcoming technologies, 5G and beyond. As we discuss later in our paper, CSI information would provide a huge amount of useful data. However, the real-time implementation and its fusion with conventional sensor data is the real concern that is not addressed. Edge computing and off-loading as the most promising solutions are rarely investigated.

There is a huge gap worth filling in this area: use of RF in SLAM, integrating variant of sensor data with RF, use of CSI in robot applications, and developing a deep learning approach, especially using the fingerprinting method.

## 8. 5G Potentials and Promises for Robot Applications

**Figure 7:**
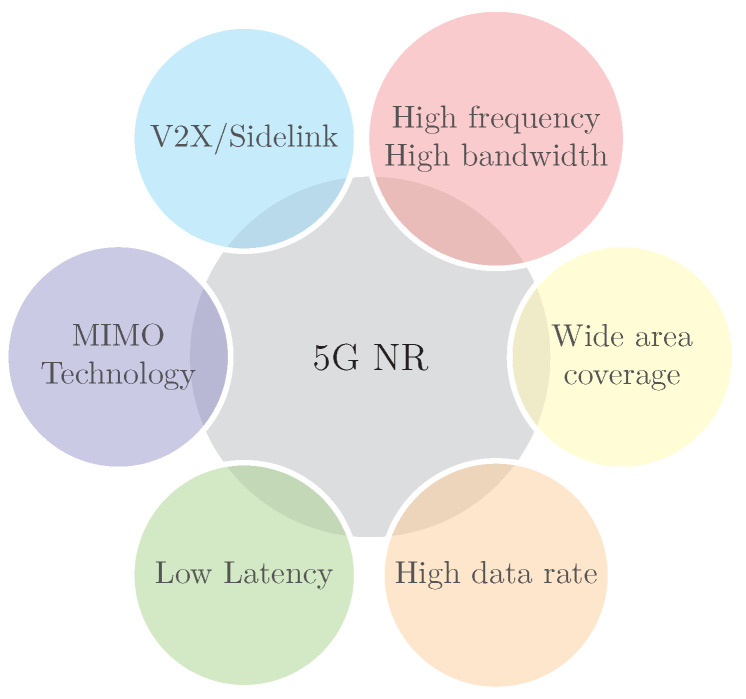
5G NR enabler for improved robot localisation.

Rolling out the 5G New Radio (NR) technology provides great potential to boost the localisation of robots and UAVs in terms of accuracy, robustness, cost, and coverage. The promising features of 5G NR for robot applications are shown in Figure 7 and include the following:

- Wide area coverage.
- MIMO technology.
- High carrier frequency.
- High bandwidth.
- Vehicle-to-Everything (V2X).
- Low latency.
- High throughput.

### 8.1. Wide Area Coverage and Inexpensive Localisation Systems

Compared to other technologies such as Wi-Fi, UWB, etc., 5G will be available almost anywhere, indoors or outdoors, since the cellular infrastructure is widely deployed in cities. Using Vehicle-to-Everything (V2X) also makes it feasible to take advantage of 5G in areas without full coverage. For example, in a collaborative scheme, parts of the device(s) or vehicle(s) can play the role of anchor or Pseudo BS for others. 5G is also considered to be an inexpensive solution because there is no specific equipment to set up as long as operating under the coverage of BS. Accordingly, taking advantage of the available infrastructure of 5G, in some cases, might remove the need for costly and energy-consuming GPS devices.

In addition, 5G confers robustness to the localisation system. Robustness is an essential feature in highly mobile scenarios, in the case where safety is of great concern. For example, consider a vehicle or a group operating in a wide area where some part of it might be GPS denied. In such a situation, relying only on GPS information may lead to failure.

### 8.2. RF Measurements with More Resolution

The 3GPP 5G New Radio (NR) is envisioned to pave the way towards achieving higher accuracy and robustness of localisation in GPS-denied environments such as indoor settings, and towards improving localisation outdoors combined with GPS. 5G NR provides improved measurements for localisation, such as time-based, angular-based, and energy-based measurements. The measurements include TOA, TDOA, AOA/AOD, and multi-cell round-trip time. For robot localisation, 5G in the downlink defines a new reference signal called a positioning reference signal (PRS), based on which these measurements can be extracted. (Readers are referred to [15] for further information).

Owing to the high carrier frequency, high bandwidth, and MIMO technology, accurate measurements will be delivered. 5G NR operates at high-frequency bands: Frequency Range 1 (FR1) (450 MHz to 6 GHz) and Frequency Range 2 (FR2) ( frequency bands from 24.25 GHz to 52.6 GHz). Relying on the Cramer-Rao Lower Bound (CRLB), the lower bound for variance of TOA is obtained by [102]:

$$\begin{array}{c} var\left(\mathrm{TOA}\right)\geq \frac{1}{8\pi^{2}BT_{s}F_{c}^{2}\mathrm{SNR}}, \end{array}$$

with *B* being the bandwidth, $F_{c}$ is the central frequency, $T_{s}$ is the duration of the signal, and SNR is signal to noise ratio. This inequality indicates that higher frequency and bandwidth contribute to higher TOA estimation accuracy.

The probability of LOS increases due to the strong path loss in higher frequencies (mmWave). This feature and the large transmission bandwidth make distinguishing between LOS and NLOS measurements in the multipath effect feasible by applying proper analysis over the received signals, resulting in highly precise time-based and angular-based localisation.

The Massive MIMO technology is one of the most noticeable enhancements and relevant features for localisation offered by 5G NR. It allows the implementation of ultra-massive antenna arrays consisting of hundreds or thousands of antennas in a single base station, leading to finer angular resolution (azimuth and elevation of the beam) of even less than one degree, which can contribute to accurate localisation.

### 8.3. Vehicle-to-Everything Standard

The Third-Generation Partnership Project (3GPP) deploys the Vehicle-to-Everything (V2X) based on Dedicated Short-Range Communications, which includes Vehicle to Vehicle (V2V), Vehicle to Network (V2N), or Vehicle to Infrastructure (V2I). The cellular V2X standard based on the 5G air interface is a fascinating feature for cooperative robotic missions, specifically through introducing the sidelink (SL), which permits vehicles to directly exchange information without other parts of the network being involved. This will play a vital role in cooperative tasks and localisation, whether the operation is within the coverage area or without BS coverage. Furthermore, the V2X feature not only allows communication of the vehicles with each other, but also with the infrastructure and even the internet. This increased connectivity improves the efficiency of the cooperative systems, enhances localisation accuracy, and makes some previously infeasible missions possible. For example, in an environment with adverse NLOS effects from the BS, which results in erroneous measurements, one or some parts of the vehicles can play the role of BSs or act as a pseudo BS.

### 8.4. Low Latency

Ultra-reliable low-latency communication (URLL) offered in 5G new radio allows for future applications which on-demand for aggressive latency for a quick reaction. The existing 4G cellular network is not appropriate for this purpose.

Low latency means a slight delay between sending and receiving information indispensable for autonomous driving and flying. For example, 1 ms minimum latency (average of 10 ms) is expected to be provided by 5G, which is a substantial breakthrough compared to the 200 ms latency typical of 4G. The central station that might perform the localisation, planning, or control must receive and send back data and command fast enough to the vehicles operating at high speed. The time for transmitting and receiving data is vital for autonomous control, where the car moves rapidly. For example, for a drone to be controlled, both localisation and control commands need to be processed and sent back to the UAV with an acceptable slight delay, resulting in a fast reaction of the UAV.

### 8.5. High Throughput

Significantly, for the uplink transmission, a high data rate is needed when offloading computations. Many algorithms try to balance computation power and accuracy, mostly because onboard computers are usually not equipped with a powerful processing unit. This concern would be prevented with offloading, which allows for implementation of complex algorithms with high accuracy in real time on a powerful server using edge computing. In addition to low latency, a high data rate is crucial for the real-time transfer of extensive sensor data, such as high-resolution images or LIDAR data. In [103], the role of edge computing and the impact of data rate in the uplink for vision-based drone navigation in the 5G network is explored. Three scenarios are tested and compared: no offloading, partial, and complete offloading. This shows how offloading can be advantageous in the network capable of providing a fast uplink rate.

### 8.6. Localisation Based on 5G

As the 5G roll-out is in the early stages, few works have investigated 5G localisation, especially for robotics applications and in a sensor fusion framework. Besides, most are solely based on simulation results, neglecting many practical aspects. For example, the impact of synchronisation error in time-based positioning or simplification of channel models is considered. In the following, we review the current state-of-the-art 5G-based localisation.

In 5G NR, pilot signals are included for positioning purposes, including Positioning Reference Signal (PRS) in the down-link and Sounded Reference Signal (SRS) in the up-link [15]. In addition to network centre frequency and bandwidth, PRS and SRS configuration also play roles in localisation accuracy. In [104], for different combinations of centre frequency, sub-carrier spacing and PRS comb-size, localisation accuracy for simple scenarios is compared in terms of Root Mean Square Error (RMSE) using simulation. The authors of [105] simulate the roadside 5G network implementation for assisted driving, showing accuracy below 20–25 cm for 50–100 MHz bandwidth. Localisation is performed based on TOA extracted as the first correlation peak between PRS and the received signal. The channel is modelled based on path loss and the TDL channel model. The impact of the geometrical placement of roadside 5G base stations on the localisation based on EKF, and how the distance from BS affects EKF linearisation error is investigated in [106]. In this paper, EKF for location estimation is also presented, in which the covariance matrix is tuned dynamically, and improvement is shown through simulation. There are several attempts at localisation in the 5G network based on CIR and CFR. The work of [107] addresses localisation in the up-link side based on 5G SRS information, in which, based on the received signal, the channel frequency response (CFR) is estimated. TOA and Direction of Arrival (DOA) are then evaluated using the well-known 2D multiple signal classification (MUSIC) algorithms. Localisation is finally performed, and an indoor experimental setup achieves accuracy of less than 1 m. The authors of [108] generated a fingerprint dataset of AOA and its corresponding amplitude based on the CSI matrix in a 5G network. Deep Neural network (DNN) was trained and used as a regressor for online estimation. Quasi Deterministic Radio channel Generator (QuaDRiGa) [109] was exploited for channel modelling. Approximately 1-meter accuracy for NLOS and 0.1 m for LOS was reported. Based on the SRS symbol, in an up-link, CFR is estimated for each base station in [110], and subsequently, TOA and AOA are jointly estimated for localisation. Accuracy below 1 m is acquired. Localisation under the fingerprinting framework is explored in [111] based on CSI, where the transfer learning concept is leveraged to reduce the real-world training effort. QuaDRiGa is leveraged to obtain synthetic CSI to pre-train the CNN model. In [112], angle-based fingerprint localisation is conducted. The fingerprints include the angles (zenith and azimuth) along with their corresponding power for all observed paths. To validate the results, simulation is performed by recreating 3D outdoor environments, including building geometry. AOA-based position estimation in a 5G network was experimentally reported in [113], where EKF was used at edge cloud for localisation. The research of [114,115] is related to receiver localisation harnessing AOA under 5G MIMO System and beam-formed RSS, respectively.

While TOA, TDOA, AOA, and RSS of the LOS path could be directly related to the relative positions of the transmitter and receiver, there is no explicit connection between the NLOS path and close distance. Thus, the localisation performance will be degraded noticeably, only relying on those measurements. In literature, NLOS error mitigation techniques and ray tracing-based approaches are carried out to compensate for the NLOS (see [116] and the references therein). Under the 5G network, this issue is addressed in [117]. The effect of NLOS in an unknown environment is dealt with in a fusion framework. Localisation and navigation are accomplished by fusing TDOA and Pedestrian Dead Reckoning (PDR). TDOA from LOS base stations is combined with TDOA from virtual Base stations placed in an unknown area whose locations are determined based on the NLOS base stations. Simulation and experiments are performed. The work of [118] goes beyond just localisation by mapping the radio environment simultaneously, taking advantage of NLOS-rich information about transmitter and receiver positions and environmental obstacles. This paper proposes joint position and orientation estimation for a mobile target and the position estimation of reflectors and scatters relying on NLOS paths. Leveraging AOA, AOD, and TOA for each NLOS path, the receiver’s location is determined only based on the received signal from one base station in 2D if there are at least three NLOS paths. It is shown in [119], in the NLOS situation, cooperation among vehicles improves situational awareness and localisation performance, as several cars operate in the same environment where they might share one or more scatters. This results in a correlated multipath structure that can contribute to the improved localisation.

## 9. Future Research Directions and Challenges

Most of the research on the use of 5G for localisation approaches the problem from the pure communication point of view, while its use in various robotic applications is still in its infancy. In this section, we shed light on future opportunities, research gaps, and challenges that will be provided by 5G for UAV and UGV applications.

### 9.1. Fingerprinting and Deep Learning Applied to CSI

Compared to range-based methods, fingerprinting has more potential to deliver higher localisation precision. On the one hand, range-based methods are limited by the accuracy of the model they apply to extract ranges or angles. On the other hand, fingerprinting may utilise the signal information to its full extent. Mainly, CSI data contain latent knowledge that can be captured by the complex AI approaches linked to fingerprinting. Hence, appropriate deep-learning models may be the key to exploiting the CSI large matrix structure effectively to hallucinate necessary abstract features for localisation. For example, deep neural networks may be able to decode these high-dimensional matrices to localise the obstacles around the receiver from the NLOS path.

Furthermore, if multi-array antennae are deployed at the receiver and transmitter, CSIs contain even richer information, since different versions of the same signal would be available as separate fingerprints. Therefore, effectively profiting from CSI information may prevent the need for several BSs for localisation. It is shown in [118] that just one BS might suffice. Lastly, in this method, the main disadvantage of the traditional RF-based method, i.e., NLOS situations, is circumvented, under which these approaches become erroneous and unreliable.

There are challenges in fingerprinting that are worth much more attention. The availability of data and the collection of enough data is not trivial. The collected dataset is not entirely reliable, as the environment constantly evolves. Transfer learning as one possible solution is suggested to exploit synthetic data or already available datasets. In this area, there still seems to be much room to investigate the integration of deep learning with CSI fingerprints.

### 9.2. Fusion of RF with Other Sensor Data

The mobility and the issue of receiving diverse information with different frequencies is a challenge that is missing in the literature. Localisation algorithms are expected to be implemented in real-time, with each measurement coming in its own time. In fingerprinting, most existing works rely on fixed target localisation and datasets consisting of RF features. However, in either the online or offline step, other sensor data such as IMU and images can be used effectively with radio-based data sets. They can either narrow down the searching area in the online phase or directly fuse with fingerprints in the offline step. From a theoretical point of view, there is still room for localisation improvement in accuracy and robustness in a data fusion scheme. For instance, to the authors’ knowledge, there is no study on fusing promising measurements provided by 5G with images, LIDAR, etc. Several combinations of the sensor data and their performance under different situations and parameters need to be researched.

### 9.3. Combination of Multiple Estimators

Combining multiple estimators for range-based localisation or fingerprinting could compensate for each method’s disadvantages, improve accuracy, and introduce resilience to failure cases. This technique is well established in the general machine-learning task and is known as ensemble learning [120]. The ensemble method combines different estimators and often yields much more accurate results than individual methods into four main paradigms Bayesian averaging, error-correcting output coding, Bagging, and boosting [121].

### 9.4. Cooperative Localisation

With V2X technology, many heterogeneous multi-robot applications comprising UGVs and UAVs can be envisioned. This enables more efficient use of robots in diverse scenarios such as coverage, formation, task distribution, etc. For example, in a search and rescue operation, a group of heterogeneous robots may collaborate, while some act as an anchor in the absence of a signal for some or all team members. On the other hand, sharing information by robots operating in the same area would endow robustness and accuracy to the localisation system, as each robot can benefit from the knowledge and estimation of its neighbour(s).

### 9.5. Orientation Estimation

Deployment of a multi-array antenna system paves the way for orientation estimation. However, attitude estimation would be challenging, especially in a 3D space while the UAV is flying. Therefore, integrating RF signals with other data to improve attitude estimation in 3D seems to be an attractive research topic which has been left untouched.

### 9.6. Experimental Setup and Realistic Simulation

Real experiments and setups for the use of 5G with robots are lacking in the state-of-the-art. This is critical, as the actual setup’s outcome does not necessarily match the predictions and expectations. Two examples are the offloading and handover in the 5G network. In the uplink, which is crucial for offloading, the data rate in 5G NR is supposed to be significantly improved, but [122] recorded the maximum of 67 Mbit/s in the uplink for a flying drone, showing no improvement compared to 4G. The impact of handover is also worth further research, especially for high-mobility scenarios. Ref. [122] shows that the handover rate between LTE and 5G is too high, which is unacceptable.

### 9.7. Off-Loading

With offloading, more computational power will be available. This calls for new complex strategies and algorithms capable of running and working in parallel. For example, the onboard computer and edge server might work collaboratively and separately. While onboard computers process some part of the data to execute a rough localisation, heavy algorithms are to be implemented on the edge server separately when there is no communication between the two processors. Both algorithms then need to be merged to output the refined localisation result. At the same time, new strategies might be developed with the processing power on edge devices to focus more on accuracy and robustness instead of the computational burden. Most state-of-the-art localisation tries to balance accuracy, complexity, and computational power.

### 9.8. Simultaneous Localisation and Mapping

A critical task in robotics is Simultaneous Localisation and Mapping (SLAM). As the robot moves in an unfamiliar environment, it needs to construct a map and localize itself on the map while navigating. The use of multiple robots instead of a single one, while each shares its maps with the others, is a fascinating topic that adds efficiency to the SLAM. Multi-robot SLAM (MRSLAM) is implemented in two ways. One involves using a central station to which all robots disseminate the collected data to carry out all the processing, construct the global map, and transmit this data back to each robot. In a decentralised system, each robot performs locally, and whenever they visit, other robots share their local map, based on which they update their map. Accordingly, one big challenge in centralised and distributed MRSLAM is the constraint on the communication bandwidth, limited computation power, and memory. 5G NR will circumvent these limitations by facilitating edge computing [123,124], providing accurate RF-based measurements, and enabling relative localisation for each vehicle in the team via sidelink and V2V technologies.

### 9.9. Vertical Localisation Accuracy

As discussed earlier, the localisation accuracy in the z-direction is always less than in x-y. Notably, the multi-path effect and the small offset of anchors in the z-direction yield poor vertical accuracy, relying on conventional methods [125]. MIMO technology and wide-band mmWave system of 5G provide promising solutions to improve the 3D positioning, such as taking full advantage of CFR in all frequencies or analysing antenna radiation pattern [126].

### 9.10. Safety

Safety is a major concern in robotics-related tasks. In general, there are two types of communication that apply to UAVs: Control and Non-Payload Communication (CNPC) and Payload Communication (PC). CNPC refers to control commands, way-points and navigation, usually in the order of several Kbps. Instead, the PC includes data transmission to the edge server, ground or aerial centre for processing. This information ranges from small-sized data, e.g., IMU, GPS, and RSS, to large-size high-resolution aerial images or LIDAR scans. While communication link reliability might not be vital for PC, avoiding communication interruptions is critical to CNPC. This leads to the concern of interruption of the CNPC link when there is no LOS path. Remarkably, using mmWave and beam-forming features of 5G call for additional care because they come with the disadvantage of very high propagation pass loss [127]. Therefore, the design of trajectory and path-planning algorithms that guarantee LOS communication would be an interesting research topic. Another concern for CNPC links is cyber-physical attacks that might corrupt data transmission or cause incorrect action of the UAVs or UGVs [39].

## 10. Conclusions

In this survey, we addressed RF-based localisation mostly from a robotic point of view. First, we explored the methods that exist for RF-based localisation extensively under two classes: Rage-based and Fingerprinting. Then, we investigated and compared current state-of-the-art RF-based localisation applied to the robotic areas. Subsequently, we discussed the challenges and solutions that 5G will provide. Finally, the future research direction was given.

5G NR will introduce features that could be harnessed to revolutionize localisation and robots’ applicability, such as low latency, high throughput, and high-resolution angular and time-based measurements. However, 5G-based localisation research is still in its infancy. Thus, many possibilities to exploit this novel communication technology in the robotic localisation context have been left unexplored. Nonetheless, the current solutions adopted for general RF receivers can be applied to 5G without modification. Until now, cameras, IMU, LIDAR, and GPS represented the predominant choice for building a SLAM system. However, the unique characteristics introduced by the 5G NR can establish RF-based localisation among the most common robotic tools for safe autonomous navigation. Leveraging such unexploited features and surpassing the main technological obstacles will be the focus of future research to ensure seamless integration of 5G into the current localisation system.

## Figures and Tables

**Table 1:** Comparison between existing reviews.

Reference	Brief Summary	1	2	3	4
[9]	A survey on RF localisation for UAV with focus on technologies and performance metrics	✓	✗	✓	✗
[8]	A survey on IoT localisation, investigating technologies and performance metrics	✗	✗	✓	✗
[10]	A comprehensive survey on localisation on WSN	✗	✗	✓	✗
[15]	A overview of the 5G-based localisation	✗	✗	✗	✓
[11]	A survey on IoT localisation	✗	✗	✓	✗
[12]	A survey on localisation techniques on WSN	✗	✗	✓	✗
[13]	A survey on localisation in WSN in 3D space	✗	✗	✓	✗
[14]	A survey on localisation on WSN algorithm and techniques	✗	✗	✓	✗
[16]	A brief review on range-free localisation algorithms in WSN	✗	✗	✓	✗
[7]	A comprehensive survey of the indoor localisation using different technologies	✗	✗	✓	✗
[17]	A brief review of technologies used for UAV positioning in indoor environment	✓	✗	✗	✗

1: UAV localisation; 2: UGV localisation; 3: Methods and algorithms; 4: 5G localisation.

**Table 2:** Comparison between range-based methods.

Range-Based Methods	Scenario	Advantages	Disadvantages
Multi-lateration Triangulation	fast and rough estimation scenarios	simple calculation	limited accuracy, sensitive to measurement error
Min-Max	fast and rough estimation scenarios	low complexity, easy implementation	limited accuracy
Multidimensional Scaling (MDS)	cooperative localisation	reduce the complexity	difficult to include the knowledge about unequal measurement error
Least Square (LS)	high accuracy	easier implementation and less demanding than ML and Bayesian, gives estimation uncertainty	computationally demanding, less optimal compared to ML and Bayesian
Maximum Likelihood (ML)	high accuracy, inaccurate prior information (outperform Bayesian)	gives estimation uncertainty	computationally demanding
Bayesian Inference	higher accuracy, sparse observations	gives estimation uncertainty	computationally demanding (more demanding than LS and ML)
Extended Kalman Filter (EKF)	real-time dynamic state estimation Easy implementation for real-time	simpler multi-sensor fusion, suitable for mobile targets, easy implementation, gives estimation uncertainty	not useful for non-Gaussian noise, less optimal compared to UKF and PF
Unscented Kalman filter (UKF)	real-time dynamic state estimation, better accuracy compared to EKF	simpler multi-sensor fusion, suitable for mobile target, gives estimation uncertainty	not useful for non-gaussian noise
Particle Filter (PF)	high accuracy dynamic state estimation	handling non-gaussian noise, gives estimation uncertainty	computationally demanding, difficult implementation

**Table 3:** Comparisons between existing works on RF-based localisation for UGVs and UAVs.

Refs	Year	Range-Based/Fingerprinting	Distributed/Centralised	Cooperative/Non-Cooperative	Anchor-Based/Anchor-Free	2D/3D	Experiment	Technique	Technology
[45]	2014	fingerprinting	centralised	non-cooperative	anchor-free	2D	Yes	PF	WLAN/RSS
[80]	2010	range-based	centralised	non-cooperative	anchor-based	2D	Yes	UKF	-/RSS
[81]	2020	range-based	centralised	non-cooperative	anchor-based	3D	Yes	UKF	UWB/TOA
[82]	2019	fingerprinting	centralised	non-cooperative	anchor-based	2D	Yes	PF	ZigBee/RSS
[83]	2021	fingerprinting	centralised	non-cooperative	anchor-free	2D	Yes	PF-ML	WiFi/RSS
[84]	2018	range-based	centralised	cooperative	anchor-based	3D	No	EKF	WiFi-UWB/RSS-TOA
[85]	2008	range-based	centralised	non-cooperative	anchor-based	2D	No	EKF	-/TDOA
[86]	2009	range-based	centralised	non-cooperative	anchor-based	2D	No	EKF	-/TDOA
[87]	2018	range-based	centralised	non-cooperative	anchor-based	3D	Yes	LS	UWB/TOA
[88]	2016	range-based	centralised	non-cooperative	anchor-based	2D	Yes	SRCKF	-/RSS
[89]	2016	range-based	distributed	cooperative	anchor-based	2D	No	DEKF	-/AOA
[90]	2020	fingerprinting	centralised	non-cooperative	anchor-free	2D	Yes	PF	-/RSS
[91]	2021	range-based	centralised	non-cooperative	anchor-based	3D	No	ML	-/RSS
[92]	2022	range-based	distributed	cooperative	anchor-based	3D	No	ML	-/RSS
[93]	2021	range-based	centralised	non-cooperative	anchor-based	3D	Yes	EKF	UWB/TW-TOF
[94]	2021	fingerprinting	centralised	cooperative	anchor-free	2D	Yes	ML	WiFi/RSS
[95]	2010	range-based	centralised	non-cooperative	anchor-based	2D	Yes	Bayesian	-/RSS
[96]	2021	range-based	centralised	non-cooperative	anchor-based	2D	Yes	LS	Cellular/AOA
[97]	2017	range-based	centralised	non-cooperative	anchor-based	3D	No	MDS-WCL	WiFi/RSS
[98]	2020	range-based	centralised	non-cooperative	anchor-based	3D	No	Lateration	-/AOA
[99]	2020	range-based	centralised	non-cooperative	anchor-based	3D	No	LS	-/TDOA-AOA
[100]	2019	range-based	centralised	non-cooperative	anchor-based	2D	Yes	RLS	UWB/TOA

## Data Availability

Not applicable.

## References

[1] Amponis G., Lagkas T., Zevgara M., Katsikas G., Xirofotos T., Moscholios I., Sarigiannidis P. (2022). Drones in B5G/6G Networks as Flying Base Stations. Drones.

[2] Azmat M., Kummer S. (2020). Potential applications of unmanned ground and aerial vehicles to mitigate challenges of transport and logistics-related critical success factors in the humanitarian supply chain. Asian J. Sustain. Soc. Responsib..

[3] Bhat S.J., Santhosh K. (2020). Is localization of wireless sensor networks in irregular fields a challenge?. Wireless Pers. Commun..

[4] Hadir A., Zine-Dine K., Bakhouya M., El Kafi J., El Ouadghiri D. (2017). Performance evaluation of DV-Hop localization algorithm for geographical routing in wireless sensor networks. Procedia Comput. Sci..

[5] Chuang P.J., Jiang Y.J. (2014). Effective neural network-based node localisation scheme for wireless sensor networks. IET Wirel. Sensor Syst..

[6] Kuriakose J., Joshi S., George V. (2014). Localization in wireless sensor networks: A survey. arXiv.

[7] Zafari F., Gkelias A., Leung K. (2019). A Survey of Indoor Localization Systems and Technologies. arXiv.

[8] Maghdid S.M. (2021). A Comprehensive Review of Indoor/Outdoor Localization Solutions in IoT era: Research Challenges and Future Perspectives. TechRxiv.

[9] Yang B., Yang E. (2021). A survey on radio frequency based precise localisation technology for UAV in GPS-denied environment. J. Intell. Robot. Syst..

[10] Chowdhury T.J., Elkin C., Devabhaktuni V., Rawat D.B., Oluoch J. (2016). Advances on localization techniques for wireless sensor networks: A survey. Comput. Netw..

[11] Khelifi F., Bradai A., Benslimane A., Rawat P., Atri M. (2019). A Survey of Localization Systems in Internet of Things. Mob. Netw. Appl..

[12] Tabassum N. (2020). Localization Techniques in Wireless Sensor Networks: A Comprehensive Survey. UGC Care J..

[13] Kumari J., Kumar P., Singh S.K. (2019). Localization in three-dimensional wireless sensor networks: A survey. J. Supercomput..

[14] Paul A.K., Sato T. (2017). Localization in wireless sensor networks: A survey on algorithms, measurement techniques, applications and challenges. J. Sens. Actuator Netw..

[15] Dwivedi S., Shreevastav R., Munier F., Nygren J., Siomina I., Lyazidi Y., Shrestha D., Lindmark G., Ernström P., Stare E. (2021). Positioning in 5G networks. arXiv.

[16] Shakshuki E., Elkhail A.A., Nemer I., Adam M., Sheltami T. (2019). Comparative Study on Range Free Localization Algorithms. Procedia Comput. Sci..

[17] Pérez M.C., Gualda D., de Vicente J., Villadangos J.M., Ureña J. (2019). Review of UAV positioning in indoor environments and new proposal based on US measurements. CEUR Workshop Proceedings.

[18] Tian Y., Tang Z., Yu Y. (2013). Third-Order Channel Propagation Model-Based Indoor Adaptive Localization Algorithm for Wireless Sensor Networks. IEEE Antennas Wirel. Propag. Lett..

[19] Martinez-Sala A., Molina-Garcia-Pardo J.M., Egea-Ldpez E., Vales-Alonso J., Juan-Llacer L., Garcia-Haro J. (2005). An accurate radio channel model for wireless sensor networks simulation. J. Commun. Netw..

[20] Lee B.H., Ham D., Choi J., Kim S.C., Kim Y.H. (2021). Genetic Algorithm for Path Loss Model Selection in Signal Strength Based Indoor Localization. IEEE Sensors J..

[21] Lin T.H., Ng I.H., Lau S.Y., Chen K.M., Huang P. A microscopic examination of an RSSI-signature-Based indoor localization system. Proceedings of the 5th Workshop on Embedded Networked Sensors (HotEmNets’08).

[22] Le H.M., Rossi J.P., Slock D. A Geometric Interpretation of Trilateration for RSS-based Localization. Proceedings of the 2020 28th European Signal Processing Conference (EUSIPCO).

[23] Norrdine A. An algebraic solution to the multilateration problem. Proceedings of the 15th International Conference on Indoor Positioning and Indoor Navigation.

[24] Booranawong A., Sengchuai K., Buranapanichkit D., Jindapetch N., Saito H. (2021). RSSI-Based Indoor Localization Using Multi-Lateration With Zone Selection and Virtual Position-Based Compensation Methods. IEEE Access.

[25] Janssen T., Berkvens R., Weyn M. (2019). Comparing machine learning algorithms for RSS-based localization in LPWAN. International Conference on P2P, Parallel, Grid, Cloud and Internet Computing.

[26] Yang K., Liang Z., Liu R., Li W. (2021). RSS-Based Indoor Localization Using Min-Max Algorithm With Area Partition Strategy. IEEE Access.

[27] Monta S., Promwong S., Kingsakda V. Evaluation of ultra wideband indoor localization with trilateration and min-max techniques. Proceedings of the 2016 13th International Conference on Electrical Engineering/Electronics, Computer, Telecommunications and Information Technology (ECTI-CON).

[28] Xie S., Hu Y., Wang Y. An improved E-Min-Max localization algorithm in wireless sensor networks. Proceedings of the 2014 IEEE International Conference on Consumer Electronics-China.

[29] Seco F., Jimenez A.R., Prieto C., Roa J., Koutsou K. A survey of mathematical methods for indoor localization. Proceedings of the 2009 IEEE International Symposium on Intelligent Signal Processing.

[30] Saeed N., Nam H., Al-Naffouri T.Y., Alouini M.S. (2019). A State-of-the-Art Survey on Multidimensional Scaling-Based Localization Techniques. IEEE Commun. Surv. Tutorials.

[31] Zhai H., Zhang Y. (2021). A recursive weighted least squares optimization algorithm based on RSS in wireless sensor networks. Internet Technol. Lett..

[32] Kang S., Kim T., Chung W. (2020). Hybrid RSS/AOA Localization using Approximated Weighted Least Square in Wireless Sensor Networks. Sensors.

[33] Li X. (2006). RSS-based location estimation with unknown pathloss model. IEEE Trans. Wirel. Commun..

[34] Sun Y., Yang S., Wang G., Chen H. (2021). Robust RSS-Based Source Localization With Unknown Model Parameters in Mixed LOS/NLOS Environments. IEEE Trans. Veh. Technol..

[35] Wang Z., Zhang H., Lu T., Gulliver T.A. (2019). Cooperative RSS-Based Localization in Wireless Sensor Networks Using Relative Error Estimation and Semidefinite Programming. IEEE Trans. Veh. Technol..

[36] Shi J., Wang G., Jin L. (2020). Least Squared Relative Error Estimator for RSS Based Localization With Unknown Transmit Power. IEEE Signal Process. Lett..

[37] Zemek R., Hara S., Yanagihara K., Kitayama K.I. A Joint Estimation of Target Location and Channel Model Parameters in an IEEE 802.15.4-based Wireless Sensor Network. Proceedings of the 2007 IEEE 18th International Symposium on Personal, Indoor and Mobile Radio Communications.

[38] Coluccia A., Ricciato F. On ML estimation for automatic RSS-based indoor localization. Proceedings of the IEEE 5th International Symposium on Wireless Pervasive Computing 2010.

[39] Mei X., Wu H., Xian J., Chen B. (2021). RSS-Based Byzantine Fault-Tolerant Localization Algorithm Under NLOS Environment. IEEE Commun. Lett..

[40] Jiang N., Zhang N. (2021). Expectation Maximization-Based Target Localization From Range Measurements in Multiplicative Noise Environments. IEEE Commun. Lett..

[41] Phoong S.Y., Ismail M.T. (2015). A comparison between Bayesian and maximum likelihood estimations in estimating finite mixture model for financial data. Sains Malays..

[42] Jin D., Yin F., Fritsche C., Gustafsson F., Zoubir A.M. (2020). Bayesian cooperative localization using received signal strength with unknown path loss exponent: Message passing approaches. IEEE Trans. Signal Process..

[43] Pearl J. (1988). Probabilistic Reasoning in Intelligent Systems: Networks of Plausible Inference.

[44] Benini A., Mancini A., Longhi S. (2013). An IMU/UWB/Vision-based Extended Kalman Filter for Mini-UAV Localization in Indoor Environment using 802.15.4a Wireless Sensor Network. J. Intell. Robot. Syst..

[45] Wu B.F., Jen C.L. (2014). Particle-Filter-Based Radio Localization for Mobile Robots in the Environments with Low-Density WLAN APs. IEEE Trans. Ind. Electron..

[46] Yin H., Xia W., Zhang Y., Shen L. UWB-based indoor high precision localization system with robust unscented Kalman filter. Proceedings of the 2016 IEEE International Conference on Communication Systems (ICCS).

[47] Wan E., Van Der Merwe R. The unscented Kalman filter for nonlinear estimation. Proceedings of the IEEE 2000 Adaptive Systems for Signal Processing, Communications, and Control Symposium (Cat. No.00EX373).

[48] Schmitz J., Schröder F., Mathar R. TDOA fingerprinting for localization in non-line-of-sight and multipath environments. Proceedings of the 2015 International Symposium on Antennas and Propagation (ISAP).

[49] Tan J., Zhao H. UAV Localization with Multipath Fingerprints and Machine Learning in Urban NLOS Scenario. Proceedings of the 2020 IEEE 6th International Conference on Computer and Communications (ICCC).

[50] Yu L., Laaraiedh M., Avrillon S., Uguen B. Fingerprinting localization based on neural networks and ultra-wideband signals. Proceedings of the 2011 IEEE International Symposium on Signal Processing and Information Technology (ISSPIT).

[51] Ha G.Y., Seo S.B., Oh H.S., Jeon W.S. LoRa ToA-based localization using fingerprint method. Proceedings of the 2019 International Conference on Information and Communication Technology Convergence (ICTC).

[52] De Sousa M.N., Thomä R.S. (2019). Applying Random Forest and Multipath Fingerprints to Enhance TDOA Localization Systems. IEEE Antennas Wirel. Propag. Lett..

[53] Wei C., Xu K., Shen Z., Xia X., Xie W., Chen L., Xu J. Joint AOA-RSS Fingerprint Based Localization for Cell-Free Massive MIMO Systems. Proceedings of the 2020 IEEE 6th International Conference on Computer and Communications (ICCC).

[54] He J., So H.C. (2020). A Hybrid TDOA-Fingerprinting-Based Localization System for LTE Network. IEEE Sensors J..

[55] Li C., Trogh J., Plets D., Tanghe E., Hoebeke J., Poorter E.D., Joseph W. CRLB-based Positioning Performance of Indoor Hybrid AoA/RSS/ToF Localization. Proceedings of the 2019 International Conference on Indoor Positioning and Indoor Navigation (IPIN).

[56] Talvitie J., Renfors M., Lohan E.S. (2015). Distance-based interpolation and extrapolation methods for RSS-based localization with indoor wireless signals. IEEE Trans. Veh. Technol..

[57] Bi J., Wang Y., Li Z., Xu S., Zhou J., Sun M., Si M. (2019). Fast Radio Map Construction by using Adaptive Path Loss Model Interpolation in Large-Scale Building. Sensors.

[58] Sun W., Xue M., Yu H., Tang H., Lin A. (2018). Augmentation of Fingerprints for Indoor WiFi Localization Based on Gaussian Process Regression. IEEE Trans. Veh. Technol..

[59] Yiu S., Dashti M., Claussen H., Perez-Cruz F. (2017). Wireless RSSI fingerprinting localization. Signal Process..

[60] Tiwary P., Pandey A., Kumar S. Differential d-Vectors for RSS based Localization in Dynamic IoT Networks. Proceedings of the 2021 International Conference on COMmunication Systems NETworkS (COMSNETS).

[61] Sun Y., Li X., Huang Z., Tian J. An Improved Closed-Form Solution for Differential RSS-based Localization. Proceedings of the 2020 IEEE Radar Conference (RadarConf20).

[62] Kjærgaard M.B. (2011). Indoor location fingerprinting with heterogeneous clients. Pervasive Mob. Comput..

[63] Fang X., Jiang Z., Nan L., Chen L. (2018). Optimal weighted K-nearest neighbour algorithm for wireless sensor network fingerprint localisation in noisy environment. IET Commun..

[64] Wang B., Gan X., Liu X., Yu B., Jia R., Huang L., Jia H. (2020). A Novel Weighted KNN Algorithm Based on RSS Similarity and Position Distance for Wi-Fi Fingerprint Positioning. IEEE Access.

[65] Zhang H., Wang Z., Xia W., Ni Y., Zhao H. (2022). Weighted Adaptive KNN Algorithm with Historical Information Fusion for Fingerprint Positioning. IEEE Wirel. Commun. Lett..

[66] Liu S., Sinha R.S., Hwang S.H. (2021). Clustering-Based Noise Elimination Scheme for Data Pre-Processing for Deep Learning Classifier in Fingerprint Indoor Positioning System. Sensors.

[67] Li X., Wang J., Liu C., Zhang L., Li Z. (2016). Integrated WiFi/PDR/Smartphone Using an Adaptive System Noise Extended Kalman Filter Algorithm for Indoor Localization. ISPRS Int. J.-Geo-Inf..

[68] Pandey A., Sequeira R., Kumar S. (2021). SELE: RSS Based Siamese Embedding Location Estimator for a Dynamic IoT Environment. IEEE Internet Things J..

[69] Wang Y., Shang Y., Tao W., Yu Y. (2021). Target Positioning Algorithm Based on RSS Fingerprints of SVM of Fuzzy Kernel Clustering. Wirel. Pers. Commun..

[70] Chriki A., Touati H., Snoussi H. SVM-based indoor localization in Wireless Sensor Networks. Proceedings of the 2017 13th International Wireless Communications and Mobile Computing Conference (IWCMC).

[71] Wye K.F.P., Zakaria S.M.M.S., Kamarudin L.M., Zakaria A., Ahmad N.B., Kamarudin K. (2021). RSS-Based Fingerprinting Localization with Artificial Neural Network. J. Phys. Conf. Ser..

[72] Burghal D., Ravi A.T., Rao V., Alghafis A.A., Molisch A.F. (2020). A comprehensive survey of machine learning based localization with wireless signals. arXiv.

[73] Sinha R.S., Hwang S.H. (2019). Comparison of CNN Applications for RSSI-Based Fingerprint Indoor Localization. Electronics.

[74] Song X., Fan X., Xiang C., Ye Q., Liu L., Wang Z., He X., Yang N., Fang G. (2019). A Novel Convolutional Neural Network Based Indoor Localization Framework With WiFi Fingerprinting. IEEE Access.

[75] El Boudani B., Kanaris L., Kokkinis A., Kyriacou M., Chrysoulas C., Stavrou S., Dagiuklas T. (2020). Implementing deep learning techniques in 5G IoT networks for 3D indoor positioning: DELTA (DeEp Learning-Based Co-operaTive Architecture). Sensors.

[76] Nagy A., Bigler T., Treytl A., Stenzl R., Wilker S., Sauter T., Wien T. RSS-based Localization for Directional Antennas. Proceedings of the 2020 25th IEEE International Conference on Emerging Technologies and Factory Automation (ETFA).

[77] Schloemann J., Buehrer R.M. (2016). On the Value of Collaboration in Location Estimation. IEEE Trans. Veh. Technol..

[78] Nazir U., Shahid N., Arshad M.A., Raza S.H. Classification of localization algorithms for wireless sensor network: A survey. Proceedings of the 2012 International Conference on Open Source Systems and Technologies.

[79] Wen C.Y., Hsiao Y.C. Decentralized anchor-free localization for wireless ad-hoc sensor networks. Proceedings of the 2008 IEEE International Conference on Systems, Man and Cybernetics.

[80] Sun C.J., Kuo H.Y., Lin C.E. A sensor based indoor mobile localization and navigation using Unscented Kalman Filter. Proceedings of the IEEE/ION Position, Location and Navigation Symposium.

[81] You W., Li F., Liao L., Huang M. (2020). Data fusion of UWB and IMU based on unscented Kalman filter for indoor localization of quadrotor UAV. IEEE Access.

[82] Luo R.C., Hsiao T.J. (2019). Dynamic Wireless Indoor Localization Incorporating With an Autonomous Mobile Robot Based on an Adaptive Signal Model Fingerprinting Approach. IEEE Trans. Ind. Electron..

[83] Wang W., Marelli D., Fu M. (2021). Dynamic Indoor Localization Using Maximum Likelihood Particle Filtering. Sensors.

[84] Goel S., Gabela J., Kealy A., Retscher G. An indoor outdoor cooperative localization framework for UAVs. Proceedings of the International Global Navigation Satellite Systems (IGNSS) Conference.

[85] Sung W., Choi S., You K. TDoA based UGV localization using adaptive Kalman filter algorithm. Proceedings of the 2008 Second International Conference on Future Generation Communication and Networking Symposia.

[86] Lee S., Lee W., You K. TDoA based UAV localization using dual-EKF algorithm. Proceedings of the International Conference on Control and Automation.

[87] Li J., Bi Y., Li K., Wang K., Lin F., Chen B.M. Accurate 3d localization for mav swarms by uwb and imu fusion. Proceedings of the 2018 IEEE 14th International Conference on Control and Automation (ICCA).

[88] Zhang W.A., Yang X., Yu L., Liu S. (2016). Sequential Fusion Estimation for RSS-Based Mobile Robots Localization With Event-Driven WSNs. IEEE Trans. Ind. Inform..

[89] Xu S., Dogançay K., Hmam H. Distributed path optimization of multiple UAVs for AOA target localization. Proceedings of the 2016 IEEE International Conference on Acoustics, Speech and Signal Processing (ICASSP).

[90] Yucel H., Elibol G., Yayan U. (2020). Wi-Fi Based Indoor Positioning System For Mobile Robots By Using Particle Filter. arXiv.

[91] Li Y., Shu F., Shi B., Cheng X., Song Y., Wang J. (2021). Enhanced RSS-Based UAV Localization Via Trajectory and Multi-Base Stations. IEEE Commun. Lett..

[92] Cheng X., Shi W., Cai W., Zhu W., Shen T., Shu F., Wang J. (2022). Communication-Efficient Coordinated RSS-Based Distributed Passive Localization via Drone Cluster. IEEE Trans. Veh. Technol..

[93] Yang B., Yang E., Yu L., Loeliger A. (2021). High-Precision UWB-Based Localisation for UAV in Extremely Confined Environments. IEEE Sensors J..

[94] Liu R., Qin Z., Zhang H., Lau B.P.L., Ismail K., Athukorala A., Yuen C., Guan Y.L., Tan U. (2021). Collaborative Radio SLAM for Multiple Robots based on WiFi Fingerprint Similarity. arXiv.

[95] Zickler S., Veloso M. RSS-based relative localization and tethering for moving robots in unknown environments. Proceedings of the 2010 IEEE International Conference on Robotics and Automation.

[96] Meles M., Rajasekaran A., Ruttik K., Virrankoski R., Jäntti R. Measurement based performance evaluation of drone self-localization using AoA of cellular signals. Proceedings of the 2021 24th International Symposium on Wireless Personal Multimedia Communications (WPMC).

[97] Stojkoska B.R., Palikrushev J., Trivodaliev K., Kalajdziski S. Indoor localization of unmanned aerial vehicles based on RSSI. Proceedings of the IEEE EUROCON 2017—17th International Conference on Smart Technologies.

[98] Al-Jazzar S.O., Jaradat Y. (2020). AOA-based drone localization using wireless sensor-doublets. Phys. Commun..

[99] Xu C., Wang Z., Wang Y., Wang Z., Yu L. (2020). Three passive TDOA-AOA receivers-based flying-UAV positioning in extreme environments. IEEE Sensors J..

[100] Nguyen T.M., Nguyen T.H., Cao M., Qiu Z., Xie L. Integrated uwb-vision approach for autonomous docking of uavs in gps-denied environments. Proceedings of the 2019 International Conference on Robotics and Automation (ICRA).

[101] Guo K., Li X., Xie L. (2020). Simultaneous cooperative relative localization and distributed formation control for multiple UAVs. Sci. China Inf. Sci..

[102] Zhang P., Lu J., Wang Y., Wang Q. (2017). Cooperative localization in 5G networks: A survey. ICT Express.

[103] Hayat S., Jung R., Hellwagner H., Bettstetter C., Emini D., Schnieders D. (2021). Edge computing in 5G for drone navigation: What to offload?. IEEE Robot. Autom. Lett..

[104] Ferre R.M., Seco-Granados G., Lohan E.S. (2019). Positioning Reference Signal Design for Positioning via 5G.

[105] Del Peral-Rosado J.A., López-Salcedo J.A., Kim S., Seco-Granados G. Feasibility study of 5G-based localization for assisted driving. Proceedings of the 2016 International Conference on Localization and GNSS (ICL-GNSS).

[106] Saleh S., El-Wakeel A.S., Noureldin A. (2022). 5G-Enabled Vehicle Positioning Using EKF With Dynamic Covariance Matrix Tuning Positionnement de véhicules à l’aide de la 5G utilisant un EKF avec réglage dynamique de la matrice de covariance. IEEE Can. J. Electr. Comput. Eng..

[107] Pan M., Liu P., Jia X., Liu S., Qi W., Huang Y. A Joint DOA and TOA Estimation Scheme for 5G Signals Under Array Modeling Errors. Proceedings of the 2021 CIE International Conference on Radar (Radar 2021).

[108] Zhang Z., Wu L., Zhang Z., Dang J., Zhu B., Wang L. AoA-and-Amplitude Fingerprint Based Indoor Intelligent Localization Scheme for 5G Wireless Communications. Proceedings of the 2021 13th International Conference on Wireless Communications and Signal Processing (WCSP).

[109] Jaeckel S., Raschkowski L., Börner K., Thiele L. (2014). QuaDRiGa: A 3-D multi-cell channel model with time evolution for enabling virtual field trials. IEEE Trans. Antennas Propag..

[110] Pan M., Liu P., Li X., Liu S., Qi W., Huang Y. A Low-Complexity Joint AOA and TOA Estimation Method for Positioning with 5G Signals.

[111] Stahlke M., Feigl T., García M.H.C., Stirling-Gallacher R.A., Seitz J., Mutschler C. Transfer Learning to adapt 6G AI-based Fingerprint Localization across Environments. Proceedings of the 2022 IEEE 95th Vehicular Technology Conference (VTC2022-Spring).

[112] Meng J., Sharma A., Tran T.X., Balasubramanian B., Jung G., Hiltunen M., Hu Y.C. A study of network-side 5G user localization using angle-based fingerprints. Proceedings of the 2020 IEEE International Symposium on Local and Metropolitan Area Networks (LANMAN).

[113] Menta E.Y., Malm N., Jäntti R., Ruttik K., Costa M., Leppänen K. (2019). On the performance of AoA–based localization in 5G ultra–dense networks. IEEE Access.

[114] Sellami A., Nasraoui L., Atallah L.N. Multi-stage localization for massive MIMO 5G systems. Proceedings of the 2020 IEEE 91st Vehicular Technology Conference (VTC2020-Spring).

[115] Klus R., Talvitie J., Valkama M. Neural network fingerprinting and GNSS data fusion for improved localization in 5G. Proceedings of the 2021 International Conference on Localization and GNSS (ICL-GNSS).

[116] Wen F., Wymeersch H., Peng B., Tay W.P., So H.C., Yang D. (2019). A survey on 5G massive MIMO localization. Digital Signal Process..

[117] Deng Z., Zheng X., Zhang C., Wang H., Yin L., Liu W. (2020). A TDOA and PDR fusion method for 5G indoor localization based on virtual base stations in unknown areas. IEEE Access.

[118] Mendrzik R., Wymeersch H., Bauch G. Joint localization and mapping through millimeter wave MIMO in 5G systems. Proceedings of the 2018 IEEE Global Communications Conference (GLOBECOM).

[119] Chu X., Lu Z., Gesbert D., Wang L., Wen X. (2021). Vehicle localization via cooperative channel mapping. IEEE Trans. Veh. Technol..

[120] Zhou Z.H. (2012). Ensemble Methods: Foundations and Algorithms.

[121] Dietterich T.G. (2000). Ensemble methods in machine learning. Proceedings of the International Workshop on Multiple Classifier Systems.

[122] Albanese A., Sciancalepore V., Costa-Pérez X. (2021). First Responders Got Wings: UAVs to the Rescue of Localization Operations in Beyond 5G Systems. IEEE Commun. Mag..

[123] Huang P., Zeng L., Chen X., Luo K., Zhou Z., Yu S. (2022). Edge Robotics: Edge-Computing-Accelerated Multi-Robot Simultaneous Localization and Mapping. IEEE Internet Things J..

[124] Huang P., Zeng L., Luo K., Guo J., Zhou Z., Chen X. ColaSLAM: Real-Time Multi-Robot Collaborative Laser SLAM via Edge Computing. Proceedings of the 2021 IEEE/CIC International Conference on Communications in China (ICCC).

[125] Charléty A., Le Breton M., Larose E., Baillet L. (2022). 2D Phase-based RFID localization for on-site landslide monitoring. Remote Sens..

[126] Sinha P., Guvenc I. (2022). Impact of Antenna Pattern on TOA Based 3D UAV Localization Using a Terrestrial Sensor Network. IEEE Trans. Veh. Technol..

[127] Hosseini N., Jamal H., Haque J., Magesacher T., Matolak D.W. UAV command and control, navigation and surveillance: A review of potential 5G and satellite systems. Proceedings of the 2019 IEEE Aerospace Conference.

